# Refining the cheatgrass–fire cycle in the Great Basin: Precipitation timing and fine fuel composition predict wildfire trends

**DOI:** 10.1002/ece3.3414

**Published:** 2017-09-25

**Authors:** David S. Pilliod, Justin L. Welty, Robert S. Arkle

**Affiliations:** ^1^ Snake River Field Station U.S. Geological Survey, Forest and Rangeland Ecosystem Science Center Boise ID USA

**Keywords:** annual forb, *Bromus tectorum*, climate, litter, sagebrush shrublands, weather

## Abstract

Larger, more frequent wildfires in arid and semi‐arid ecosystems have been associated with invasion by non‐native annual grasses, yet a complete understanding of fine fuel development and subsequent wildfire trends is lacking. We investigated the complex relationships among weather, fine fuels, and fire in the Great Basin, USA. We first modeled the annual and time‐lagged effects of precipitation and temperature on herbaceous vegetation cover and litter accumulation over a 26‐year period in the northern Great Basin. We then modeled how these fine fuels and weather patterns influence subsequent wildfires. We found that cheatgrass cover increased in years with higher precipitation and especially when one of the previous 3 years also was particularly wet. Cover of non‐native forbs and native herbs also increased in wet years, but only after several dry years. The area burned by wildfire in a given year was mostly associated with native herb and non‐native forb cover, whereas cheatgrass mainly influenced area burned in the form of litter derived from previous years’ growth. Consequently, multiyear weather patterns, including precipitation in the previous 1–3 years, was a strong predictor of wildfire in a given year because of the time needed to develop these fine fuel loads. The strong relationship between precipitation and wildfire allowed us to expand our inference to 10,162 wildfires across the entire Great Basin over a 35‐year period from 1980 to 2014. Our results suggest that the region's precipitation pattern of consecutive wet years followed by consecutive dry years results in a cycle of fuel accumulation followed by weather conditions that increase the probability of wildfire events in the year when the cycle transitions from wet to dry. These patterns varied regionally but were strong enough to allow us to model annual wildfire risk across the Great Basin based on precipitation alone.

## 1 INTRODUCTION

Wildfire frequencies have increased in many arid and semi‐arid regions of the world, partly because of changes in climate, vegetation, and land use (Brooks et al., 2004; Krawchuk, Moritz, Parisien, Van Dorn, & Hayhoe, 2009; Dennison, Brewer, Arnold, & Moritz, 2014). Desert shrublands are historically fuel limited because sparse perennial bunchgrasses and shrubs compete for limited water and other resources, resulting in barren interspaces among plants maintained, in part, by biological soil crusts (Reisner, Grace, Pyke, & Doescher, 2013). Livestock grazing and other soil disturbances have facilitated invasion of these interspaces by annual species, changing the amount and continuity of fine fuels in some shrublands (Davies & Nafus, 2013; Leffler, Monaco, James, & Sheley, 2016; Reisner et al., 2013). Greater road access and human use of desert environments have helped spread invasive plants and created new wildfire ignition sources (Mann et al., 2016; Pyke, Chambers, Beck, Brooks, & Mealor, 2016; Van Linn et al., 2013). A positive feedback between fire‐prone non‐native annual grasses and wildfire has resulted in a grass–fire cycle in many arid and semi‐arid environments around the world (D'Antonio & Vitousek, 1992). This combination of increased fine fuel, fuel continuity, and ignitions, coupled with climate drivers, has resulted in more fire starts, larger fires, longer fire seasons, and shorter fire return intervals (Abatzoglou & Kolden, 2011).

The Great Basin, the largest cold desert in North America (505,772 km^2^), is a prime example of a shrubland ecosystem, that is changing rapidly because of increased size and frequency of wildfires. The semi‐arid climate of the Great Basin supports vast salt desert scrub (e.g., *Atriplex* spp.) and sagebrush (e.g., *Artemisia* spp.) shrublands. However, there is considerable evidence that invasion of cheatgrass (*Bromus tectorum*) and other non‐native annual species has led to a grass–fire cycle that has increased fire frequency in the northern Great Basin up to four times historic levels (Balch, Bradley, D'antonio, & Gómez‐Dans, 2013) and steadily transformed native shrubland habitats into cheatgrass‐dominated grasslands (Brooks et al., 2004; D'Antonio & Vitousek, 1992). This conversion can occur after a single fire but is especially likely after repeated fires (Chambers et al., 2014). The altered fire regimes and loss of sagebrush habitats in the Great Basin have threatened many native species and fostered ambitious conservation strategies for protecting remaining habitat from wildfire and restoring native shrublands after fire (Arkle et al., 2014; Coates et al., 2016; Knick, Dobkin, Rotenberry, Schroeder, & Vander Haegen, 2003).

Non‐native annual grasses and forbs in the Great Basin are often most successful in hotter, drier locations and after disturbances that remove perennial grasses, alter biological soil crusts, or otherwise expose soils (Haubensak, D'Antonio, & Wixon, 2009). Part of this competitive advantage relates to differences in life history traits between non‐native annual and native perennial grasses (Alba, Skálová, McGregor, D'antonio, & Pyšek, 2015). For example, cheatgrass, which originated from Eurasia and arrived in North America in the late 1800s, is successful within the Great Basin by germinating earlier (i.e., early fall through early spring), growing faster (including at cooler temperatures), and producing more seed than native perennials (James, Drenovsky, Monaco, & Rinella, 2011; Mack & Pyke, 1983). That rapid growth and high densities of cheatgrass enable it to compete effectively with native perennials for limited resources like water and nutrients, particularly in shallow soils (James et al., 2011). Cheatgrass produces viable seed by late spring and early summer (Chambers, Roundy, Blank, Meyer, & Whittaker, 2007; Chambers et al., 2016; Novak & Mack, 2001) well before many native grasses and forbs. These life history strategies result in early senescence, which creates a dry, spatially continuous fuel bed during the hottest, driest part of the year when wildfires in the Great Basin are most common (Davies & Nafus, 2013). When summer wildfires burn through areas infested with cheatgrass, the exposed soil creates an ideal environment for cheatgrass seed to germinate upon the arrival of fall and winter precipitation. The rapid vegetative growth of cheatgrass suppresses growth and recovery of native perennial plants, particularly in parts of the Great Basin where precipitation and native perennial bunchgrass cover are low (Brummer et al., 2016). Hence, the propensity of cheatgrass to dominate following fire is highest in warmer, drier locations (Taylor, Brummer, Rew, Lavin, & Maxwell, 2014) as well as where prefire biological soil crust cover is low and native perennial grasses and forbs are depleted (Chambers et al., 2014; Shinneman & Baker, 2009).

Interannual variability in germination, growth, establishment, and biomass of grassland and shrubland plants can be substantial and is related to both precipitation and temperature (Clarke, Latz, & Albrecht, 2005; Holmgren et al., 2006; Horn, Bishop, & Clair, 2017; Horn, Nettles, & Clair, 2015; Hsu & Adler, 2014; Sala, Gherardi, Reichmann, Jobbagy, & Peters, 2012). In the Great Basin, adequate precipitation and soil moisture in the fall or winter and spring are critical for germination and growth of annual grasses, particularly when temperatures are favorable (Bradley, Curtis, & Chambers, 2016; Mack & Pyke, 1983). Hence, years with above‐average precipitation generally have higher cover and biomass of cheatgrass (Chambers et al., 2007). As this biomass senesces and dries, it accumulates as litter, which can persist over several years. Litter helps entrap the next generation of annual plant seed and can further promote annual plant establishment (Jones, Chambers, Johnson, Blank, & Board, 2015). Consequently, high cheatgrass biomass has been observed 2 years after an unusually wet winter and spring (Bradley & Mustard, 2005). The combination of high annual grass biomass and multiyear accumulation of litter may greatly increase the probability of wildfire and drive the grass‐fire cycle of the Great Basin (Balch et al., 2013). This pattern may be reversed, however, during extended periods of drought when many annual species have low germination and survival (Bradley et al., 2016).

**Figure 1:**
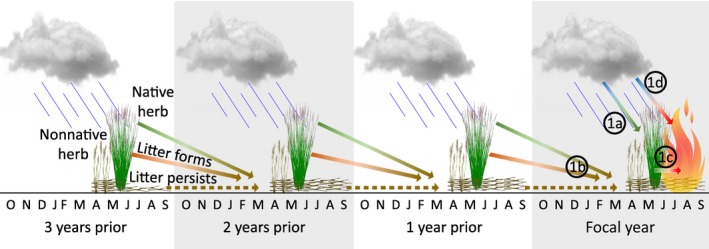
Conceptual model of hypothesized relationships among weather, fine fuel, and fire. Fine fuel is characterized as herbaceous non‐native and native vegetation, and litter. Litter is shown as dark brown plant matter lying horizontally, which can persist across years. Numbered circles correspond to a subset of our study objectives (1a–d) examined at a focal study area within the northern Great Basin. Objective 1a refers to the relationship between weather and herbaceous fine fuel, 1b refers to the relationship between herbaceous plant cover in previous years and litter cover in a focal year, 1c refers to the relationship between herbaceous fine fuel and fire, and 1d refers to the relationship between weather and fire. Objectives 2 and 3 (not shown) were examined across the entire Great Basin, with Objective 2 addressing the relationship between weather and fire at the Great Basin‐scale, and Objective 3 assessing our ability to use this relationship to predict and forecast relative fire risk across this area

Despite considerable evidence that the cheatgrass–fire cycle has led to a new fire regime in the Great Basin (Balch et al., 2013; Brooks et al., 2004; D'Antonio & Vitousek, 1992), a complete understanding of how fine fuels develop and influence wildfire patterns is lacking. Our study has three objectives aimed at advancing understanding of the effects of multiyear weather on fine fuels and wildfire in the Great Basin (Figure 1). Objective 1 was to assess the relationships among weather, herbaceous vegetation, and annual wildfire characteristics in a northern Great Basin landscape using long‐term (26 year) field data. Objective 2 was to examine the relationship between weather and annual wildfire characteristics across the entire Great Basin over the last 35 years. Objective 3 was to demonstrate how these analyzes could create a spatially explicit wildfire risk assessment based on weather data alone. Our hypotheses were as follows: (1) higher than normal winter and spring precipitation increase herbaceous plant cover, particularly cheatgrass, and other non‐native annual species (Hsu, Powell, & Adler, 2012; Rao & Allen, 2010; Robinson et al., 2013); (2) higher annual cheatgrass cover is associated with more wildfires and larger area burned (D'Antonio & Vitousek, 1992); and (3) years with above‐average antecedent precipitation, especially 1–2 years prior, result in more wildfires and more area burned (Balch et al., 2013; Billings, 1994; Knapp, 1998; Littell, McKenzie, Peterson, & Westerling, 2009). These hypotheses were relevant for each of our objectives, but we were only able to test the first two hypotheses in our focal study area in the northern Great Basin where long‐term vegetation data were available. Spatially continuous vegetation data are unavailable on an annual basis across the entire Great Basin at this time (but see Boyte & Wylie, 2016), and thus, an additional goal of this study was to determine if interpolated, spatially continuous, monthly weather data could predict wildfire patterns and wildfire risk across this vast landscape in the absence of annual fuel load data.

## 2 MATERIALS AND METHODS

### 2.1 Study area

**Figure 2:**
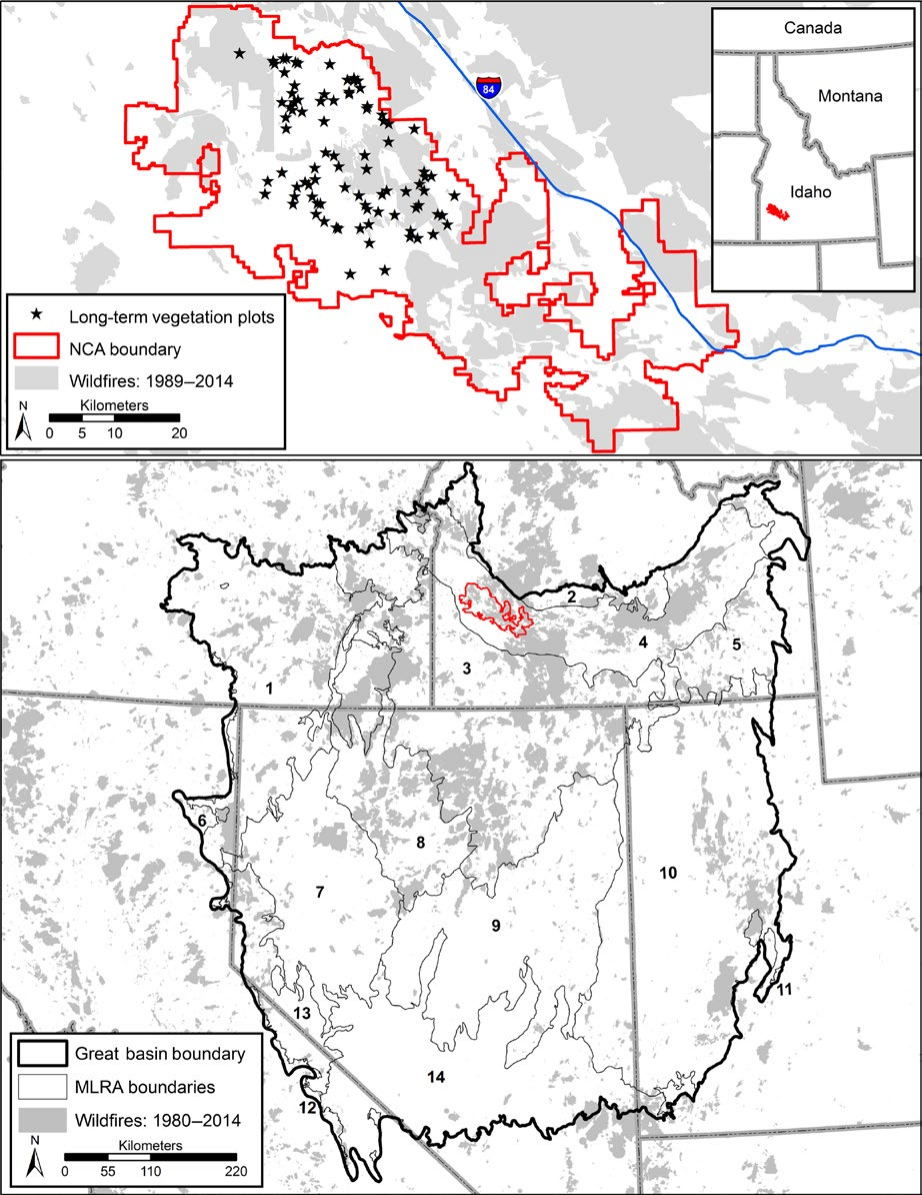
Focal and Great Basin study area maps. Historic fire polygons are shown in gray. The focal study area is located at the Morley Nelson Snake River Birds of Prey National Conservation Area located within the Snake River Plains Major Land Resource Area (MLRA) of the northern Great Basin. Long‐term vegetation plots are shown. The Great Basin study area is delineated as three Ecoregions (see text) and subdivided by Major Land Resource Areas (MLRA) numbered: (1) Malheur High Plateau, (2) Central Rocky and Blue Mountain Foothills, (3) Owyhee High Plateau, (4) Snake River Plains, (5) Eastern Idaho Plateaus, (6) Klamath and Shasta Valleys and Basins, (7) Fallon‐Lovelock Area, (8) Humboldt Area, (9) Central Nevada Basin and Range, (10) Great Salt Lake Area, (11) Wasatch and Uinta Mountains, (12) Sierra Nevada Mountains, (13) Carson Basin and Mountains, and (14) Southern Nevada Basin and Range. The location of the focal study area is shown for reference (red polygon)

We defined the Great Basin based on three U.S. EPA Level III Ecoregions (Snake River Plain, Northern Basin and Range, and Central Basin and Range; http://www.epa.gov/wed/pages/ecoregions/level_iii_iv.htm), which include parts of eastern California, southern Idaho, Nevada, southeastern Oregon, and western Utah (Figure 2). We subdivided the Great Basin study area using the U.S. Department of Interior Bureau of Land Management's (BLM) Major Land Resource Area (MLRA) boundaries because they provided a spatial scale more amenable to our analyzes than would Level IV Ecoregions (which were too finely divided) and because MLRAs are used by BLM, the primary fire management agency in the region.

**Figure A1:**
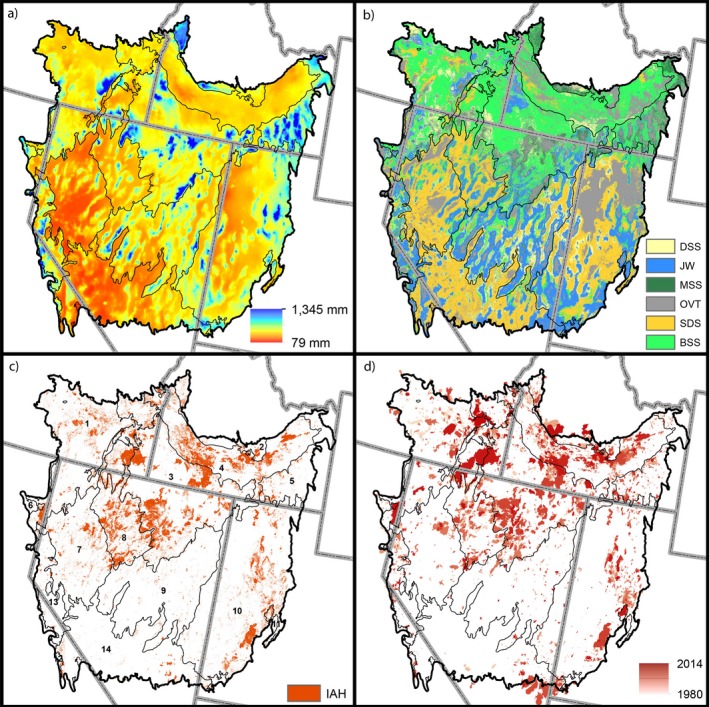
Great Basin study area showing (a) 1980–2014 average annual precipitation, (b) potential vegetation types reclassified from LANDFIRE (see Table A1), (c) areas dominated by introduced annual herbaceous vegetation (IAH) according to LANDFIRE. Major Land Resource Area (MLRA) codes in the lower right panel are: (1) Malheur High Plateau, (2) Central Rocky and Blue Mountain Foothills, (3) Owyhee High Plateau, (4) Snake River Plains, (5) Eastern Idaho Plateaus, (6) Klamath and Shasta Valleys and Basins, (7) Fallon‐Lovelock Area, (8) Humboldt Area, (9) Central Nevada Basin and Range, (10) Great Salt Lake Area, (11) Wasatch and Uinta Mountains, (12) Sierra Nevada Mountains, (13) Carson Basin and Mountains, (14) Southern Nevada Basin and Range, (d) fires burned from 1980 to 2014

The Great Basin is characterized by basin and range topography with elevations ranging from 341 to 4,340 m. Annual precipitation ranges from 79 to 1,291 mm (Appendix 1: Fig. A1a) and falls mainly as winter snow and early‐spring rain. Average daily minimum and maximum temperatures range from −4.7 to 8.6°C in winter (October–March) and 9.8–28.4°C in summer (July–September), with both precipitation and temperature varying strongly with elevation and latitude. The dominant plant communities, as characterized by LANDFIRE's potential vegetation type (www.landfire.gov), include Wyoming sagebrush (*Artemisia tridentata wyomingensis*) shrublands (26% of the land area), Salt desert shrublands (23%), juniper (*Juniperus* spp.) woodlands (18%), dwarf sagebrush (*Artemisia arbuscula*) shrublands (8%), and mountain sagebrush (*Artemisia tridentata vaseyana*) shrublands (4%; Appendix 1: Fig. A1b). Other vegetation types, such as riparian, aspen (*Populus tremuloides*), and mixed conifer forests, occupy the remaining land area (21%). Habitats most strongly influenced by the grass–fire cycle include sagebrush shrublands, juniper woodlands, salt desert shrublands, and grasslands. Invasive annual grasslands, predominantly composed of cheatgrass, are now widespread (Appendix 1: Fig. A1c).

Within the Snake River Plains MLRA located in the northern Great Basin, we assessed annual changes in herb and litter cover in a focal study area where a long‐term (26‐year) vegetation dataset was available. These long‐term monitoring plots were established in 1989 with the goal of tracking trends in vegetation at the Orchard Combat Training Center located in the Morley Nelson Snake River Birds of Prey National Conservation Area in southwestern Idaho (NCA; Figure 2). The NCA is a 1,963 km^2^ landscape of intact and invaded (by non‐native grasses and forbs) sagebrush steppe and salt desert scrub ecological sites. Like the majority of the Great Basin, the focal study area experiences some cattle and sheep grazing, usually in the winter and spring. Elevations across the NCA range from 687 to 1,110 m. Average daily minimum and maximum temperatures range from −2.3 to 9.4 °C in winter (October–March) and 11.8 to 30.8°C in summer (July–September). Total annual precipitation ranges from 172 to 321 mm, with over 60% falling as rain and snow during the winter and nearly 30% as rain in the spring (April–June).

Wildfires in the Great Basin burn in the late spring and summer, and occasionally into the fall (Brooks, Matchett, Shinneman, & Coates, 2015). On average, wildfire ignitions occur between 14 May and 9 October each year, but fire ignitions have occurred as early as 5 March and as late as 23 November (1984–2014, *n* = 2,593 fires; Monitoring Trends in Burn Severity; www.mtbs.gov, accessed 7 July 2017). The average start date of fires in the Great Basin is 30 July. Start dates vary little among different MLRAs in the Great Basin, but the length of fire season tends to be shorter in the south compared with the north (Brooks et al., 2015). The Snake River Plain, located in the northern Great Basin, has the longest fire season (111 days). The average annual fire start date within the NCA (i.e., within our focal study area within the Snake River Plain) is 12 July, but fires have been observed as early as 5 June and as late as 1 October (1984–2014; *n* = 77 fires; MTBS).

### 2.2 Field sampling

We assessed annual changes in herb cover at 57 permanent plots in the NCA. In 1989, a permanently marked, 100‐m transect was established in each of these randomly placed plots. Each plot has been measured annually since using line‐point intercept sampling (Elzinga, Salzer, & Willoughby, 1998). All species present at each point were recorded every meter along each transect. Points extend vertically from ground to the highest canopy, and data were recorded for each species contacted across multiple canopy layers. Canopy cover for each species and functional group was calculated at each plot by dividing the number of points where a species or functional group was present by 100. Using this approach, we created the following vegetation/fine fuel variables: Cheatgrass (cover of the non‐native annual *Bromus tectorum*); ExoticForb (cover of all non‐native forbs, which were dominated by curveseed butterwort—commonly called burr buttercup, *Ceratocephala testiculata*; prickly Russian thistle, *Salsola tragus*; clasping pepperweed, *Lepidium perfoliatum*; tall tumblemustard, *Sisymbrium altissimum*); NativeHerb (cover of all native herbs, which was dominated by Sandberg bluegrass, *Poa secunda*, a common species at drier sites in the Great Basin; Holthuijzen & Veblen, 2015); and Litter (all non‐native and native herbaceous litter covers).

### 2.3 Weather variable development

For our focal landscape analyzes in the northern Great Basin (objective 1), we used raw precipitation (mm) and temperature (°C) values derived from monthly PRISM data (PRISM, 2010). PRISM data are interpolated values from hundreds of meteorological stations and reported as 800‐m gridded monthly data (Daly, Gibson, Taylor, Johnson, & Pasteris, 2002). We used these data to calculate variables representing seasonal weather in each year 1989–2014 for each pixel intersecting a plot in our focal study area (Table 1). In total, we generated 77 precipitation and five temperature variables for each season and year at each plot (see Table 1). For precipitation, we calculated seasonal: (1) precipitation within a given year, (2) average and maximum precipitation 1, 2, and 3 years prior, and (3) the change in precipitation from 1 to 3 years prior to a given year. We calculated the change in precipitation from 1, 2, and 3 years previous to a given year as year being calculated minus the average of the 3 years prior, the year being calculated minus the average of the 2 years prior, and the year being calculated minus 1 year prior. We repeated this procedure using the maximum (instead of average) precipitation from the previous 3 years and the previous 2 years. The difference values calculated from these procedures (regardless of number of years included or whether average or maximum was used) allowed us to assess how different the precipitation was during a given year relative to the previous 1–3 years. Increasingly negative values indicated that a given year was drier than preceding years and increasingly positive values indicated an increase in precipitation relative to previous years. Values close to zero indicated little change in precipitation relative to previous years. Seasons are defined in Table 1 and are consistent with seasonal precipitation characterization in the Great Basin (Bates, Svejcar, Miller, & Angell, 2006) and cluster analyzes of monthly precipitation in other deserts of the region as well (Tagestad, Brooks, Cullinan, Downs, & McKinley, 2016).

**Table 1:** Precipitation and temperature variables used in analyzes

Variable component	Description
*Precipitation and temperature time periods*	
ANNUAL	Water year (October–September)
WINTER	Winter (October–March)
SPRING	Spring (April–June)
WIN+SPR	Winter–spring (October–June)
SUMMER	Summer (July–September)
*Precipitation and temperature time lags*	
P	Total precipitation during specified time period in the given year
1yrP	Total precipitation during specified time period 1 year prior to the given year
2yrPave	Average precipitation during specified time period 2 years prior to the given year
2yrPmax	Maximum precipitation during specified time period 2 years prior to the given year
3yrPave	Average precipitation during specified time period 3 years prior to the given year
3yrPmax	Maximum precipitation during specified time period 3 years prior to the given year
Tave	Average daily temperature during specified time period in the given year
*Precipitation difference from previous years*	
Δ1yrP	Delta precipitation from 1 year prior relative to the given year (given year − previous year)
Δ2yrPave	Delta precipitation from the average of 2 years prior relative to the given year (given year—average of 2 years previous)
Δ2yrPmax	Delta precipitation from the maximum of 2 years prior relative to the given year (given year—maximum of 1–2 years previous)
Δ3yrPave	Delta precipitation from the average of 3 years prior relative to the given year (given year—average of 3 years previous)
Δ3yrPmax	Delta precipitation from the maximum of 3 years prior relative to the given year (given year—maximum of 1–3 years previous)

Precipitation variables combined three variable components: (1) time period, (2) time lags, and (3) difference from previous year(s). Given year refers to any water year (October–September) from 1980 to 2014 for which these calculations were made.

At the scale of the Great Basin (objectives 2 and 3), we used an anomaly approach for our precipitation variables because of the considerable regional variation across such a large landscape. We did not include temperature in these Great Basin‐wide models because temperature was not an important predictor in any of the focal study area models. We converted each precipitation variable from units of mm to an anomaly by dividing each variable by its 65‐year (1950–2014) average value and multiplying by 100. Thus, we expressed each pixel's value for a given variable in a given year (1980–2014) as a percentage, with values >100% representing above‐average and values <100% representing below‐average precipitation for that pixel in that year.

### 2.4 Fire variable development

We compiled fire perimeter data from multiple sources (e.g., Monitoring Trends in Burn Severity (MTBS), GeoMac, BLM offices, USDA Forest Service regions, and state fire agencies) to create, to our knowledge, the most comprehensive inventory of fire perimeter data available (Appendix 1: Fig. A1d; Welty, Pilliod, & Arkle, 2017). The dataset consists of over 57,000 wildfires that occurred from 1878 to 2015 in the U.S., the vast majority of which are more recent (e.g., post‐1980, due to increased reporting) and from the western U.S., due to the prevalence of public land and ecosystems with frequent fire return intervals. To create this dataset, we used GIS to merge all contributing fire perimeter datasets and dissolve overlapping fires within a given year (see Welty et al., 2017). We attempted to prevent duplication of a given fire in our dataset by assuming: (1) that an area can only burn once per year (to remove overlapping polygons representing the same fire); and (2) that burned polygons <1 km apart in the same year are part of the same fire or fire complex, whereas those >1 km apart are separate fires (allows for multipart perimeters within 1 km, but prevents distant fires from being merged). Thus, our dataset could underrepresent the number of fires and hectares burned in any given year when an area truly burned more than once in the same year (unlikely in the Great Basin). We might also underrepresent the number of fires in a year when truly different fires or fire complexes are within 1 km of one another. Finally, we would omit truly unburned islands or misrepresent fire perimeters whenever a coarser, more generalized version of a fire perimeter is larger than that of a smaller version of the same fire (e.g., figure 2 in Balch et al., 2013). However, systematic data checks revealed these caveats had minimal influence on final fire counts or area burned. For analyzes pertaining to the present study, we only include the 10,162 fires occurring within our Great Basin study boundary from 1980 to 2014 to limit regional and temporal biases due to differences in fire record submission (see Appendix 1: Fig. A1d for fire polygons used in analyzes). Variables representing annual fire characteristics were calculated for each year by summing burned area (haBURNED), obtaining a count of fires (nFIRES), and by defining uncharacteristically “large” fire years (BIGYR1SD) as those years when haBURNED was >1 *SD* from the long‐term mean for the area being evaluated.

### 2.5 Data analysis

We subdivided Objective 1 into four parts (Figure 1). We first assessed the relative importance of precipitation and temperature for fine fuel loads (Objective 1a), specifically cheatgrass, non‐native forbs, native herbs, and litter cover, over the 26 years of data. We then assessed which plant functional groups drive litter cover in subsequent years and the number of years over which litter from these different groups may persist (Objective 1b). Next, we assessed the relationship between herbaceous fine fuels and annual fire characteristics (Objective 1c). Finally, we examined the indirect relationship between weather (i.e., seasonal and annual precipitation and temperature, including time lags of 1–3 years) and annual fire characteristics (Objective 1d), without considering fine fuel loads (Figure 1). This final relationship was important to establish so that we could extend our analyzes across the Great Basin (see objective 2) where spatially continuous, annual weather data existed, but annual vegetation data did not. We modeled the relationships among precipitation, temperature, herb cover, and fire characteristics using nonparametric multiplicative regression (NPMR, see below).

For objective 2, we evaluated weather as a driver of fire characteristics across the Great Basin using NPMR to model how annual variability in precipitation influences the number of fires, area burned, and the probability of a uncharacteristically large fire year within MLRAs of the Great Basin. We used zonal statistics to calculate averages for all 77 precipitation variables within each MLRA and year (sample unit = MLRA‐year). We used this approach, rather than calculating precipitation conditions within the actual fire polygons in each MLRA because the latter approach would result in different locations in each year contributing to average values for each MLRA, thus confounding the effects of location (within each MLRA) and year (our variable of interest in this analysis). We calculated the within‐MLRA averages to represent, for each year: (1) precipitation in that year, (2) precipitation 1, 2, and 3 years prior, and (3) the change in precipitation from 1 to 3 years prior relative to the given year. Response variables were calculated for each MLRA‐year by summing haBURNED, nFIRES, and by determining which MLRA‐years had BIGYR1SD.

To address objective 3, we developed a NPMR model predicting wildfire occurrence within 800‐m pixels. We used GIS to determine the centroid pixel of all 10,162 wildfires and to randomly select 21,858 unburned pixels, a value that summed to 32,000 pixels, the computational limit of our analysis software. For each wildfire pixel, we extracted all 77 of our precipitation variables for the year in which the wildfire occurred. For each randomly selected (unburned) pixel, we extracted all 77 of our precipitation variables for a randomly selected year between 1980 and 2014. Thus, this analysis evaluated how precipitation conditions at wildfire sites (in the year of the wildfire) differ from those at unburned locations in the same range of years. We then applied this model to the observed precipitation values for each pixel in the Great Basin (*n* = 1.2 million 800‐m pixels) for each of the recent years 2011–2013 and mapped the resulting fire risk estimates to illustrate spatial and temporal variability in risk across sequential years in the Great Basin. We used the “sm” package in R Studio version 0.99.489 (Bowman & Azzalini, 2014; R Development Core Team, 2014) to create probability density distributions of the estimated fire risk values for all pixels that intersected a fire polygon (burned pixels) and all pixels that did not intersect a fire polygon (unburned pixels) in each year 2011–2013. We plotted these distributions and used 100 bootstrap runs (sampling with replacement) to test the null hypothesis that for each year 2011–2013, there was no difference in the distribution of estimated fire risk values for burned and unburned pixels. We provide a *p*‐value for each of the 3 years evaluated.

Nonparametric multiplicative regression models were run using HyperNiche 2.28 software (McCune & Mefford, 2009). We used this approach because it allowed us to assess how predictor variables interacted multiplicatively (rather than additively) and potentially nonlinearly (see Arkle et al., 2014 for details). For each NPMR analysis, we used a local linear model (LLR; for models with quantitative response variables) or a local mean model (LMM; for models with binary response variables) with Gaussian weighting functions to conduct free‐search iterations of combinations of predictors (screened to remove correlated variables) and their tolerances (tolerance = *SD* of Gaussian weighting function of each predictor) to maximize model fit, while minimizing overfitting (McCune, 2009). Fit for models with binary response variables was assessed using logβ, which evaluates the improvement of the fitted model over the naïve model (i.e., the overall occupancy rate), expressed in powers of 10. Fit of models with quantitative response variables was assessed using cross‐validated *R* ^2^ (*xR* ^2^). We controlled overfitting through minimum average neighborhood size, minimum data‐to‐predictor ratio, and “leave‐one‐out” cross‐validation of logβ and *xR* ^2^. Bootstrap resampling (each dataset resampled with replacement 100 times) was used to quantify the stability of models (when different combinations of sample units were analyzed) by providing an average logβ or *xR* ^2^ (±*SE*). We also report the average neighborhood size (N* = average number of sample units contributing to the estimate of the response at each point on the modeled surface) and Monte Carlo randomization results (null hypothesis = fit of best model is no better than chance, using the same number of predictor variables in 100 free‐search iterations with randomly shuffled response values). For each predictor variable in each final model, we report sensitivity (a measure of relative importance of quantitative predictors) and tolerance (a measure of niche breadth). Sensitivity indicates the relative importance of quantitative predictors, where a value of 1 indicates that, on average, changing the value of the predictor by ±5% of its range, results in a 5% change in the response estimate. This provides a measure of relative importance for each quantitative predictor in the model. High tolerance values, relative to the range of the predictor, indicate that data points farther in predictor space are used to estimate response values at the target point.

We showed three‐dimensional graphical representations of each model to illustrate interactions among the most influential variables, with a few exceptions. On two occasions, we showed the first and third most important variables (based on sensitivity values): (1) Litter response to precipitation in our focal study area and (2) nFires response to precipitation in our focal study area. In both cases, the second most important variable was redundant with the top variable. We showed two‐dimensional representations of models when a single variable had more than twice the relative importance of the next most important variable in the model.

## 3 RESULTS

### 3.1 Relationships among weather, herbaceous vegetation, and wildfire in a northern Great Basin landscape

We present the results of Objective 1 in four parts: (1) weather predicts herbaceous vegetation and litter cover (fine fuels); (2) herbaceous vegetation predicts litter cover in subsequent years; (3) fine fuels predict wildfire characteristics; and (4) weather predicts wildfire characteristics.

#### 3.1.1 Objective 1a: Weather predicts herbaceous vegetation and litter cover (fine fuels)

Temperature and precipitation varied year‐to‐year across the 26 years of field sampling in our focal study area in the northern Great Basin. From 1989 to 2014, at our study plots, mean seasonal temperatures ranged from 1.5 to 5.0°C (winter), 11.6 to 16.4°C (spring), and 18.1 to 23.2 C (summer). Over this time period, annual (water year) precipitation at our plots ranged from 169 to 381 mm (26‐year average ± *SD* = 269 ± 65 mm/year).

**Figure 3:**
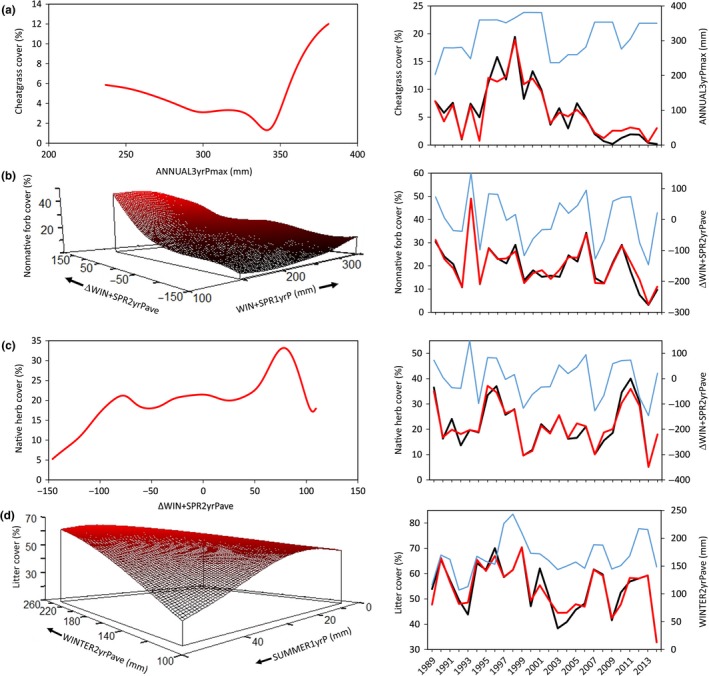
Modeled relationships between precipitation (mm) and (a) cheatgrass, (b) non‐native forb, (c) native herb, and (d) herbaceous litter cover from 57 plots sampled annually 1989–2014 in sagebrush ecological sites at the Morley Nelson Snake River Birds of Prey National Conservation Area in southwestern Idaho. Negative values of delta precipitation variables (e.g., ΔWIN + SPR2yrPave) indicate seasons that were drier than corresponding seasons in previous years. Panels at right show observed (black lines) and model estimated (red lines) cover values (primary *y*‐axes) and observed values of the most influential precipitation variable from each model (blue lines; secondary y‐axes) through time

Across years, average canopy cover of cheatgrass ranged 0.2%–19.4%. This variability was associated with annual precipitation, but not temperature. Cheatgrass cover (Cheatgrass) was highest in years with high precipitation (ANNUALP; not shown), particularly when at least one of the previous 3 years had very high precipitation (ANNUAL3yrPmax; Figure 3a). ANNUAL3yrPmax was a very influential variable indicating that this previous precipitation likely created the seed bank that then was available for germination 1–3 years later. Cheatgrass cover also tended to be higher the first year that winter–spring precipitation transitioned from dry to wet (i.e., positive values of ΔWIN + SPR2yrPave; not shown). This three‐variable model explained 69% of the interannual variability in cheatgrass cover (*p *=* *.024; Table 2).

**Table 2:** Nonparametric multiplicative regression (NPMR) analysis results for models using precipitation variables^a^ to predict fine fuel cover^b^ in a focal study area of the northern Great Basin from 1989 to 2014

Response	Model Fit (*xR* ^2^)	N*	Bootstrap (mean fit ± *SD*)	% Improvement	Predictor	Sensitivity	Tolerance
Cheatgrass	0.69	5.3	0.97 ± 0.04	12.5	ANNUAL3yrPmax	2.4	17 (10%)
					ANNUALP	1.1	169 (80%)
					ΔWIN+SPR2yrPave	1	178 (60%)
ExoticForb	0.74	1.8	0.95 ± 0.03	5.4	ΔWIN+SPR2yrPave	0.6	59.4 (20%)
					WIN+SPR1yrP	0.45	39.3 (20%)
					WINTER2yrPmax	0.39	20.3 (15%)
NativeHerb	0.63	2.1	0.88 ± 0.02	11.5	ΔWIN+SPR2yrPave	1.9	14 (5%)
					WIN+SPR2yrPmax	0.8	118 (75%)
					SPRING3yrPmax	0.6	55 (45%)
Litter	0.65	2.6	0.91 ± 0.01	5.6	WINTER2yrPave	0.8	54 (40%)
					WINTER1yrP	0.7	14 (10%)
					SUMMER1yrP	0.3	13 (30%)

N* is the average neighborhood size or the average number of sample units contributing to the estimate of the response at each point on the modeled surface. *xR* ^2^ is the cross‐validated *R* ^2^. Percent Improvement is the increase in model fit (as measured by *xR* ^2^) obtained by adding the final variable to each model. Large improvements indicate that increased model complexity is warranted because of improved model fit.

a See Table 1 for definitions of precipitation variables.

b Cheatgrass = cheatgrass (*Bromus tectorum*) cover, ExoticForb = non‐native forb cover, NativeHerb = native herbaceous cover, and Litter = herbaceous litter cover.

Non‐native forb cover (ExoticForb) was relatively high, on average up to 25.4%, but also variable through time. The four species that mostly comprised ExoticForb varied across the 26‐year period: tall tumblemustard (0.2%–11.9%), clasping pepperweed (0.07%–12.3%), burr buttercup (0.6%–13.1%), and prickly Russian thistle (0.01%–21.7%). This variability was associated with seasonal precipitation, but not temperature. Cover of non‐native forbs was highest in years with winters and springs that were wetter than the average of the previous 2 years (i.e., positive values of ΔWIN + SPR2yrPave), especially when the previous winter–spring was particularly dry (i.e., low values of WIN + SPR1yrP; Figure 3b). Similarly, non‐native forb cover was higher when at least one of the previous two winters was particularly dry (WINTER2yrPmax; not shown). This three‐variable model explained 74% of the interannual variability in non‐native forb cover (*p *=* *.073; Table 2).

Native herb cover (NativeHerb), which included all native grasses and forbs, was strongly associated with seasonal precipitation, but not temperature. The dominant native herbaceous vegetation was the perennial bunchgrass Sandberg bluegrass, which ranged 4%–34% cover annually and averaged 17.5% across all years. No other native perennial grass or forb had cover greater than 2% on average. Native herb cover was highest the first wet year following 2 years with low winter–spring precipitation (i.e., positive values of ΔWIN+SPR2yrPave; Figure 3c). Native herb cover also was higher when at least one of the previous 2 years had dry winters and springs (i.e., low values of WIN + SPR2yrPmax; not shown) and especially when at least one of those springs was particularly dry (i.e., low values of SPRING3yrPmax; not shown). This three‐variable model explained 63% of interannual variability in native herb cover (*p *=* *.073; Table 2).

Litter cover (Litter) varied annually, but with periods of accumulation over consecutive years. Across years, average litter cover was 54%. Sixty‐five percent of the interannual variability in litter cover could be predicted by an interaction of two variables: average winter precipitation for the previous 2 years (WINTER2yrPave) and precipitation the previous summer (SUMMER1yrP) (*p *=* *.073; Table 2). Litter cover was highest when the two previous winters and the previous summer had high precipitation, but relatively low when previous winters were dry and the previous summer was wet (Figure 3d). Litter cover was not related to temperature.

#### 3.1.2 Objective 1b: Herbaceous vegetation predicts litter cover in subsequent years

**Figure 4:**
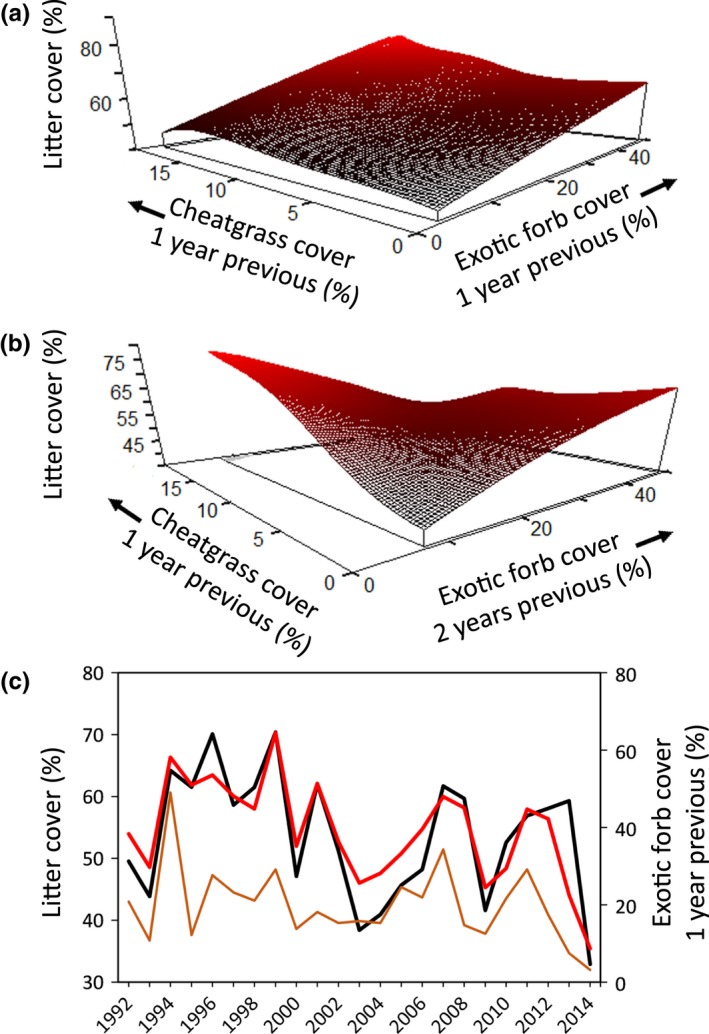
(a, b) Modeled relationships between plant cover in previous years and litter cover from 57 plots sampled annually 1992–2014 in sagebrush ecological sites at the Morley Nelson Snake River Birds of Prey National Conservation Area in southwestern Idaho. Data from 1989 to 1991 could not be included because this analysis required plant cover data collected 1–3 years prior to litter cover data. (c) Observed (black line) and model estimated (red line) cover values (primary *y*‐axis) and observed values of the most influential vegetation variable (brown line; secondary *y*‐axis) through time

Using cover of cheatgrass (Cheatgrass), non‐native forbs (ExoticForb), and native herbs (NativeHerb) in a given year and 1–3 years previous as potential predictors, we found that herbaceous litter cover (Litter) was best predicted by an interaction between non‐native forb and cheatgrass cover 1 year earlier, as well as non‐native forb cover 2 years earlier (*xR* ^2^ = 0.53; *p *=* *.024; N* = 7.3; 67.3% improvement over the best model with one fewer predictor variable). Therefore, litter cover in any given year was positively associated with non‐native forbs and cheatgrass, but with a 1‐ to 2‐year time lag (Figure 4). Litter cover was not related to cover of plant functional groups growing in that year nor was it strongly predicted by Cheatgrass, ExoticForb, or NativeHerb cover from 3 years prior.

#### 3.1.3 Objective 1c: Fine fuels predict wildfire characteristics

**Figure 5:**
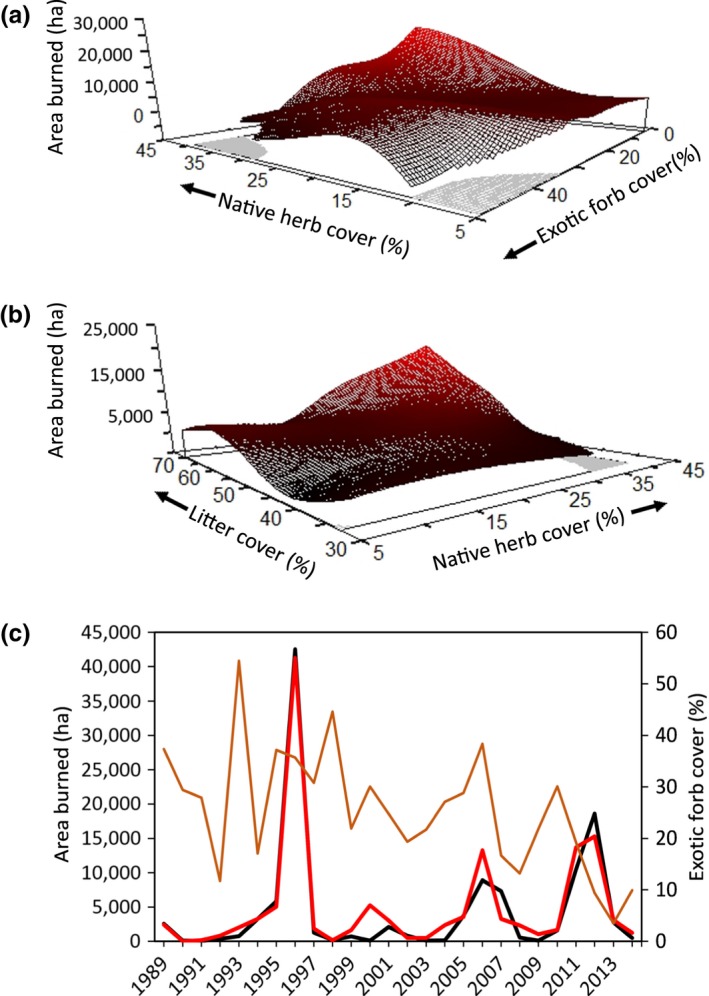
(a, b) Modeled relationships between fine fuels and area burned annually in sagebrush ecological sites at the Morley Nelson Snake River Birds of Prey National Conservation Area in southwestern Idaho. Fine fuels were characterized from vegetation data collected from 57 plots sampled annually 1989–2014. Gray areas are regions of predictor space with too few observations to make reliable estimates of area burned. (c) Observed (black line) and model estimated (red line) cover values (primary *y*‐axis) and observed values of the most influential vegetation variable (brown line; secondary *y*‐axis) through time

**Figure 6:**
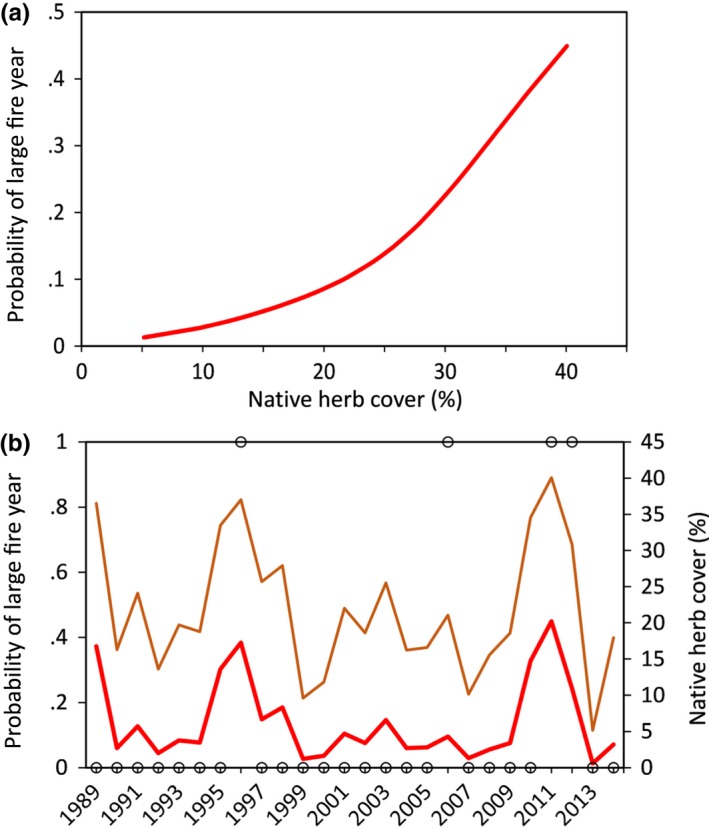
(a) Modeled relationship between fine fuels in sagebrush ecological sites at the Morley Nelson Snake River Birds of Prey National Conservation Area in southwestern Idaho and uncharacteristically large fire years (BIGYR1SD, see text for definition). Fine fuels were characterized from vegetation data collected from 57 plots sampled annually 1989–2014. (b) Observed large fire years (black circles) and model estimated (red line) probability of large fire year (primary *y*‐axis), along with observed values of the most influential vegetation variable (brown line; secondary *y*‐axis) through time

The number of fires (nFIRES) and area burned (haBURNED) annually in our focal study area varied substantially. However, interannual variability in the number of fires was not predictable based on herbaceous plant nor litter cover, as no combination of predictor variables produced a model with an *xR* ^2^ > 0.05. The area burned annually ranged from 56 to 42,543 ha (average ± *SD* = 4,448 ± 8,873 ha/year) and was associated with herbaceous plant and litter cover in a given year (*xR* ^2^ = 0.40; *p *=* *.024; Table 3). Specifically, the area burned was positively related to native herb and litter cover, and negatively related to non‐native forb cover present in that year (Figure 5). Four years fell outside of the first standard deviation for haBURNED and were consequently classified as uncharacteristically large fire years for our binary analysis of BIGYR1SD. Large fire years were more likely in years when native herb cover was high (Figure 6). The probability of a large fire year increased to 0.40 as native herb cover surpassed 40% (log β = 0.152; *p *=* *.019; Table 3).

**Table 3:** Nonparametric multiplicative regression (NPMR) analysis results for models using fine fuel cover^a^ to predict wildfire characteristics^b^ in a focal study area of the northern Great Basin from 1989 to 2014

Response	Model Fit	Fit Metric	N*	Bootstrap (mean fit ± *SD*)	% Improvement	Predictor	Sensitivity	Tolerance
nFIRES	na	*xR* ^2^	na	na	na	na	na	na
haBURNED	0.40	*xR* ^2^	1.9	0.95 ± 0.10	302	ExoticForb	0.66	9.17 (20%)
						Litter	0.52	7.50 (20%)
						NativeHerb	0.52	6.99 (20%)
BIGYR1SD	0.15	logβ	11.4	0.14 ± 0.03	na	NativeHerb	0.39	6.90 (20%)

N* is the average neighborhood size or the average number of sample units contributing to the estimate of the response at each point on the modeled surface. *xR* ^2^ is the cross‐validated *R* ^2^. Percent Improvement is the increase in model fit (as measured by *xR* ^2^) obtained by adding the final variable to each model. Large improvements indicate that increased model complexity is warranted because of improved model fit.

a ExoticForb = non‐native forb cover, NativeHerb = native herbaceous cover, and Litter = herbaceous litter cover.

b nFIRES = number of fires within the focal study area in a given year, haBURNED = area burned within the focal study area in a given year, BIGYR1SD = binary variable indicating whether the ha burned within the focal study area in a given year was >1 *SD* from the mean.

#### 3.1.4 Objective 1d: Weather predicts wildfire characteristics

**Figure 7:**
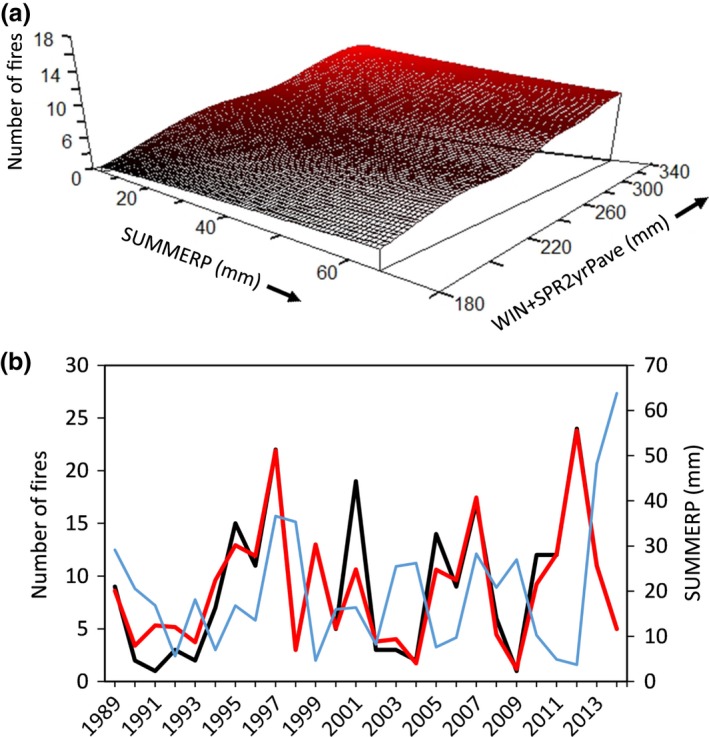
(a) Modeled relationships between precipitation (mm) and the number of fires occurring annually at the Morley Nelson Snake River Birds of Prey National Conservation Area in southwestern Idaho, 1989–2014. (b) Observed (black line) and model estimated (red line) number of fires (primary *y*‐axis) and observed values of the most influential precipitation variable (blue line; secondary *y*‐axis) through time

The number of fires that burned annually was associated with precipitation, but not temperature. The number of fires was highest when the preceding two winters and springs were particularly wet (i.e., higher values of WIN + SPR2yrPave) and when the summer of the fire year was dry (i.e., lower values of SUMMERP; Figure 7). Dry summers within the previous 2 years (SUMMER2yrPmax) also explained the number of fires that burned in our focal study area (not shown) and the three‐variable model explained 69% of the variation in number of fires across 26 years (*p *=* *.073; Table 4).

**Table 4:** Nonparametric multiplicative regression (NPMR) analysis results for models using precipitation variables^a^ to predict fire characteristics^b^ in a focal study area of the northern Great Basin from 1989 to 2014

Response	Model fit	Fit metric	N*	Bootstrap (mean fit ± *SD*)	% Improvement	Predictor	Sensitivity	Tolerance
nFIRES	0.69	*xR* ^2^	3.4	0.94 ± 0.01	86.2	SUMMERP	1.0	45 (75%)
						SUMMER2yrPmax	0.8	4 (10%)
						WIN+SPR2yrPave	0.8	31 (20%)
haBURNED	0.38	*xR* ^2^	1.6	0.70 ± 0.10	286.1	ANNUALP	1.6	10 (5%)
						ΔSPRING3yrPave	0.4	35 (25%)
BIGYR1SD	2.54	logβ	1.5	3.61 ± 2.27	24.0	SPRING2yrPave	1.5	4 (5%)
						WIN+SPR1yrP	0.2	70 (20%)

N* is the average neighborhood size or the average number of sample units contributing to the estimate of the response at each point on the modeled surface. *xR* ^2^ is the cross‐validated *R* ^2^. Percent Improvement is the increase in model fit (as measured by *xR* ^2^) obtained by adding the final variable to each model. Large improvements indicate that increased model complexity is warranted because of improved model fit.

a See Table 1 for definitions of precipitation variables.

b nFIRES, number of fires within the focal study area in a given year; haBURNED, area burned within the focal study area in a given year; BIGYR1SD, binary variable indicating whether the ha burned within the focal study area in a given year was >1 *SD* from the mean.

The area burned annually across our focal study area could be predicted by an interaction of annual precipitation (ANNUALP) and spring precipitation relative to the average spring precipitation over the 3 years prior to the fire (ΔSPRING3yrPave). Although this model explained 38% of interannual variability in area burned, the model fit was not better than that obtained from any of the two‐predictor models with randomly shuffled response values (*p *=* *.75; Table 4). Several years with extremely high burn totals may have influenced model fit. However, predicting uncharacteristically large fire years (BIGYR1SD) was difficult as well, with the best model (SPRING2yrPave and ΔWIN + SPR1yrP) fitting no better than randomly shuffled data 31% of the time (*p *=* *.31; Table 4).

### 3.2 Relationship between weather and annual wildfire characteristics across the entire Great Basin over the last 35 years

**Figure 8:**
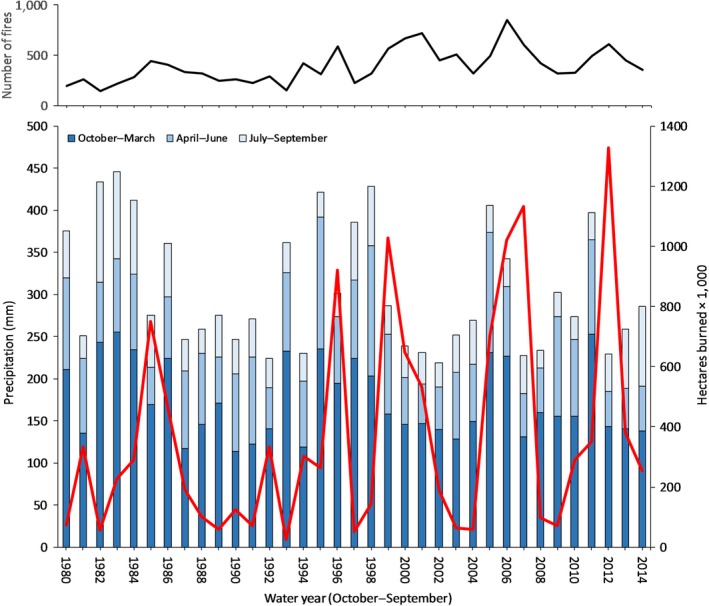
Within the Great Basin, average winter (October–March), spring (April–June), and summer (July–September) precipitation (primary *y*‐axis) and total area burned by wildfires (ha * 1,000; secondary *y*‐axis) from 1980 to 2014. Upper panel shows the number of reported fires in the Great Basin annually

Across the Great Basin, annual precipitation was highly variable (Figure 8). Few years from 1980 to 2014 are within the first standard deviation of the mean precipitation, indicating that receiving an “average” amount of precipitation is a rare occurrence. Moreover, Great Basin precipitation generally follows a multiyear wet and multiyear dry pattern (Figure 8). We found that, on average, the Great Basin experienced 2.5 years of uninterrupted positive and 2.5 years of uninterrupted negative precipitation anomaly. These patterns were not always synchronous across all regions (i.e., MLRAs) within the Great Basin, as some years showed strong latitudinal variation.

**Figure 9:**
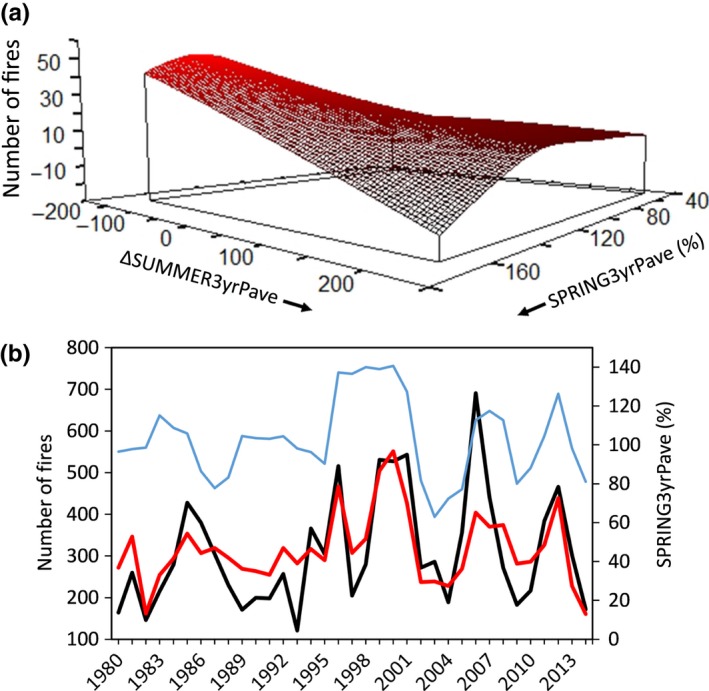
(a) Modeled relationship between precipitation anomaly (percent of average) and the number of fires occurring annually within MLRAs of the Great Basin from 1980 to 2014. Negative values of ΔSUMMER3yrPave indicate summers that were drier than the average of the previous three summers. Negative *y*‐axis values occur due to a high rate of change in the response variable in that region of predictor space. (b) Observed (black line) and model estimated (red line) number of fires across the Great Basin (primary *y*‐axis) and observed values of the most influential precipitation variable (blue line; secondary *y*‐axis) through time

The number of fires in a given MLRA‐year was highest when the preceding three springs were wetter on average (i.e., high values of SPRING3yrPave) and when the summer of the fire year was much drier than the preceding three summers (i.e., negative values of ΔSUMMER3yrPave; *p *=* *.02; Table 5; Figure 9). This model explained 66% of the variation in the annual number of fires across the 14 MLRAs from 1980 to 2014. However, this relationship was weaker in some MLRAs (e.g., Great Salt Lake) and stronger in others, such as the Owyhee High Plateau (Appendix 1: Fig. A2Aa,b). Winter precipitation variables were not important predictors of the number of fires nor were precipitation variables derived solely from a single year.

**Table 5:** Nonparametric multiplicative regression (NPMR) analysis results for models using precipitation variables^a^ to predict fire characteristics^b^ across the Great Basin from 1980 to 2014

Response	Model fit	Fit metric	N*	Bootstrap (mean fit ± *SD*)	% Improvement	Predictor	Sensitivity	Tolerance
nFIRES	0.66	*xR* ^2^	14.8	0.68 ± 0.06	8.7	MLRA	na	na
						SPRING3yrPave	0.19	17.8 (15%)
						ΔSUMMER3yrPave	0.14	263.0 (70%)
haBURNED	0.43	*xR* ^2^	15.8	0.53 ± 0.08	5.2	MLRA	na	na
						ΔSPRING1yrP	0.17	51.1 (15%)
						WINTER1yrP	0.11	124.0 (65%)
						SPRING3yrPave	0.09	89.4 (75%)
BIGYR1SD	13.80	log β	39.6	15.80 ± 2.90	4.0	ΔSPRING1yrP	0.44	17.1 (5%)
						ΔANNUAL1yrP	0.21	20.1 (10%)
						ΔWINTER3yrPave	0.05	51.0 (20%)

Major land resource area (MLRA) represents bioclimatic regions.

a See Table 1 for definitions of precipitation variables.

b nFIRES, number of fires across the Great Basin in a given year; haBURNED, area burned across the Great Basin in a given year; BIGYR1SD, binary variable indicating whether the ha burned across the Great Basin in a given year was >1 *SD* from the mean.

**Figure 10:**
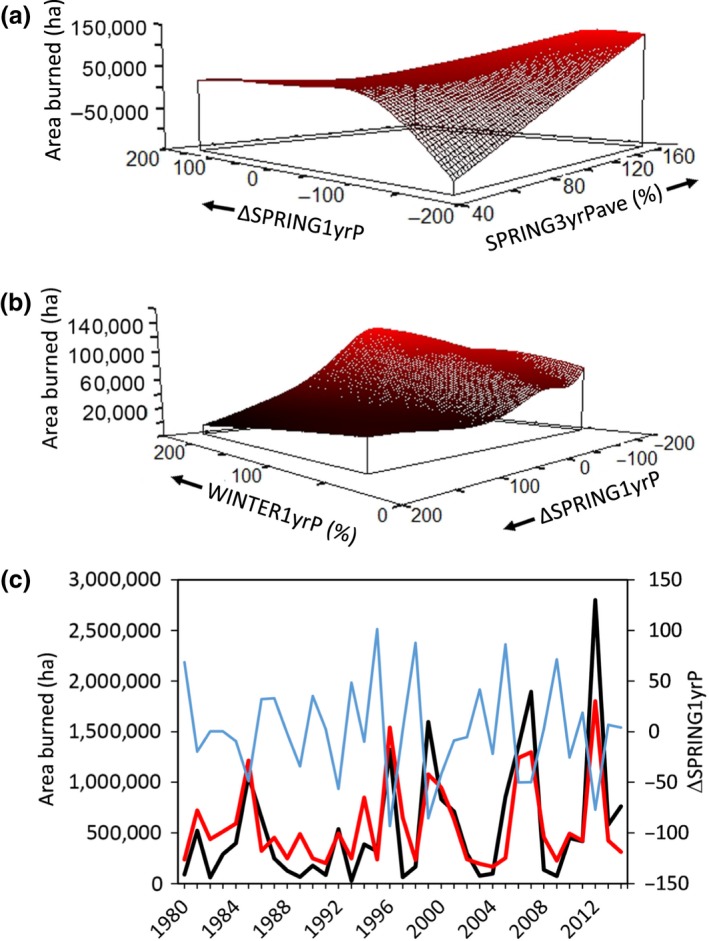
(a, b) Modeled relationship between precipitation anomaly (percent of average) and area burned annually within MLRAs of the Great Basin from 1980 to 2014. Negative values of ΔSPRING1yrP indicate springs that were drier than the previous spring. Negative *y*‐axis values occur due to a high rate of change in the response variable in that region of predictor space. (c) Observed (black line) and model estimated (red line) area burned across the Great Basin (primary *y*‐axis) and observed values of the most influential precipitation variable (blue line; secondary *y*‐axis) through time

**Figure A4:**
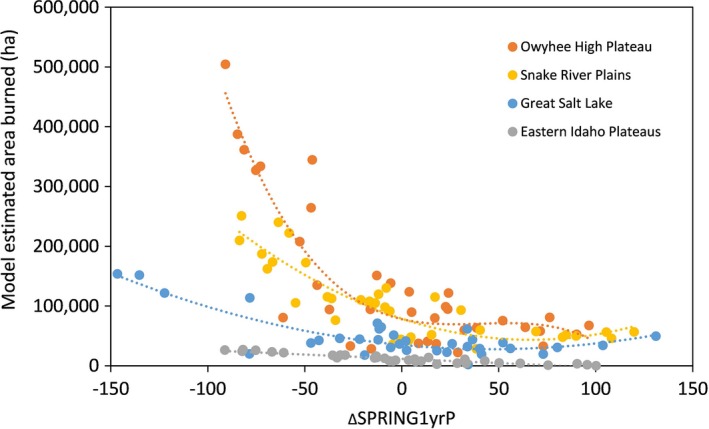
Model estimated area burned versus one‐year change in spring precipitation for four representative MLRAs. Each point is one year (1980–2014) in a given MLRA. Model estimates are derived from precipitation variables in the Great Basin‐level model

The area burned in a given MLRA‐year was highest when the preceding three springs were wetter on average (i.e., high values of SPRING3yrPave), when the preceding winter was wetter than normal (i.e., high values of WINTER1yrP), and when the spring of the fire year was much drier than the preceding spring (i.e., negative values of ΔSPRING1yrP; *p *=* *.02; Table 5; Figure 10). This model explained 43% of the variation in area burned across the Great Basin from 1980–2014. Analysis of residuals indicated that the model fit observed data well (Appendix 1: Fig. A3a,b). Most of the unexplained variation in area burned in any given MLRA was caused by a few (1–3) years with exceptionally large fire years. The effects of these variables on area burned varied strongly with MLRA (Appendix 1: Fig. A4). Precipitation variables derived solely from a given year were not important predictors of the area burned in that year.

**Figure 11:**
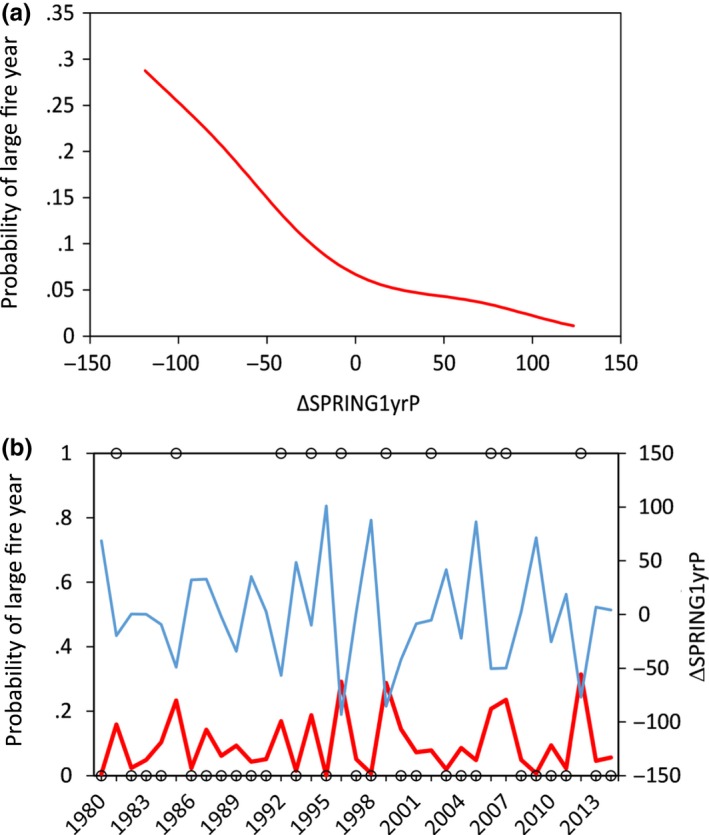
(a) Modeled relationship between precipitation anomaly (percent of average) and uncharacteristically large fire years (BIGYR1SD, see text for definition) in the Great Basin. Negative values of ΔSPRING1yrP indicate springs that were drier than the previous spring. (b) Observed large fire years (black circles) and model estimated (red line) probability of large fire year (primary *y*‐axis), along with observed values of the most influential precipitation variable (blue line; secondary *y*‐axis) through time

The probability of an uncharacteristic large fire year, a year when total area burned in an MLRA exceeded 1 *SD* of the 35‐year average for that MLRA (BIGYR1SD), was higher when spring precipitation was unusually low compared to the year before (i.e., negative values of ΔSPRING1yrP; Figure 11). Large fires also were more likely when a dry year followed a wet year (i.e., negative values of ΔANNUAL1yrP), especially when winter precipitation was low relative to the average winter precipitation the 3 years before the fire (i.e., negative values of ΔWINTER3yrPave; Table 5). The ΔSPRING1yrP was the most influential variable, with a value of −100 resulting in a 30% chance of an MLRA experiencing a fire year outside of 1 *SD* of the 35‐year average (Figure 11).

### 3.3 A spatially explicit wildfire risk assessment based entirely on precipitation data across the Great Basin

**Figure A5:**
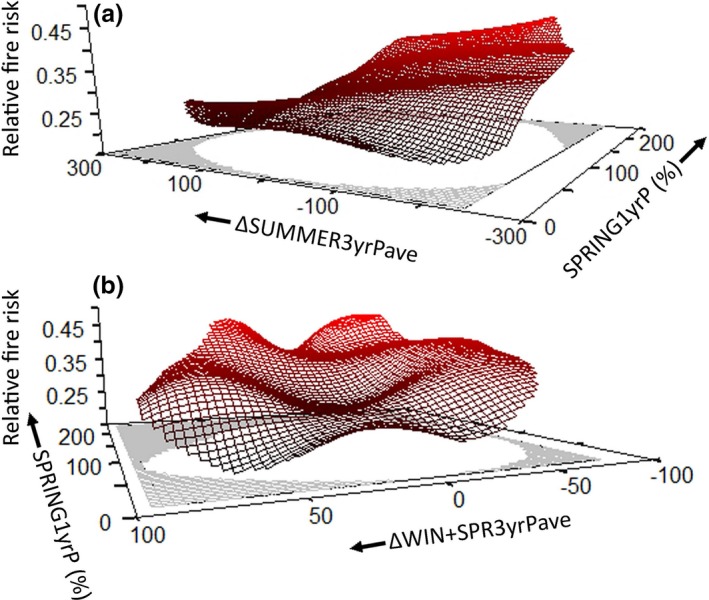
Modeled relationships between precipitation and annual relative fire risk across the Great Basin from 1980 to 2014. The best fitting model contained three precipitation predictor variables: ΔSUMMER3yrPave, SPRING1yrP, and ΔWIN + SPR3yrPave. Panel (a) shows that fire risk is highest the year following wet springs and particularly when summers are dry (negative values) compared to the three years prior. Panel (b) shows that fire risk is highest the year following wet springs and particularly when winter and spring precipitation in the given year is lower than the three years prior

Precipitation conditions at wildfire centroid pixels (in the year of the wildfire) differed from conditions at unburned (in any year) pixel locations (*p *=* *.04; logβ = 186.6; N* = 1,859; 17.1% improvement over the best model with one fewer predictor variable; Appendix 1: Fig. A5). Wildfire centroids tended to occur in locations that experienced a wet spring during the preceding year (i.e., high values of SPRING1yrP; Sensitivity = 0.36, Tolerance = 17.5 [5% of predictor's range]), had summer precipitation that was drier than the average of the three previous years (i.e., negative values of ΔSUMMER3yrPave; Sensitivity = 0.09, Tolerance = 88.9 [15% of predictor's range]), and had winter–spring precipitation that was drier than the average of the three previous years (i.e., negative values of ΔWIN + SPR3yrPave; Sensitivity = 0.44, Tolerance = 16.6 [5% of predictor's range]). Probability of fire occurrence was most sensitive to ΔWIN + SPR3yrPave and least sensitive to ΔSUMMER3yrPave.

**Figure 12:**
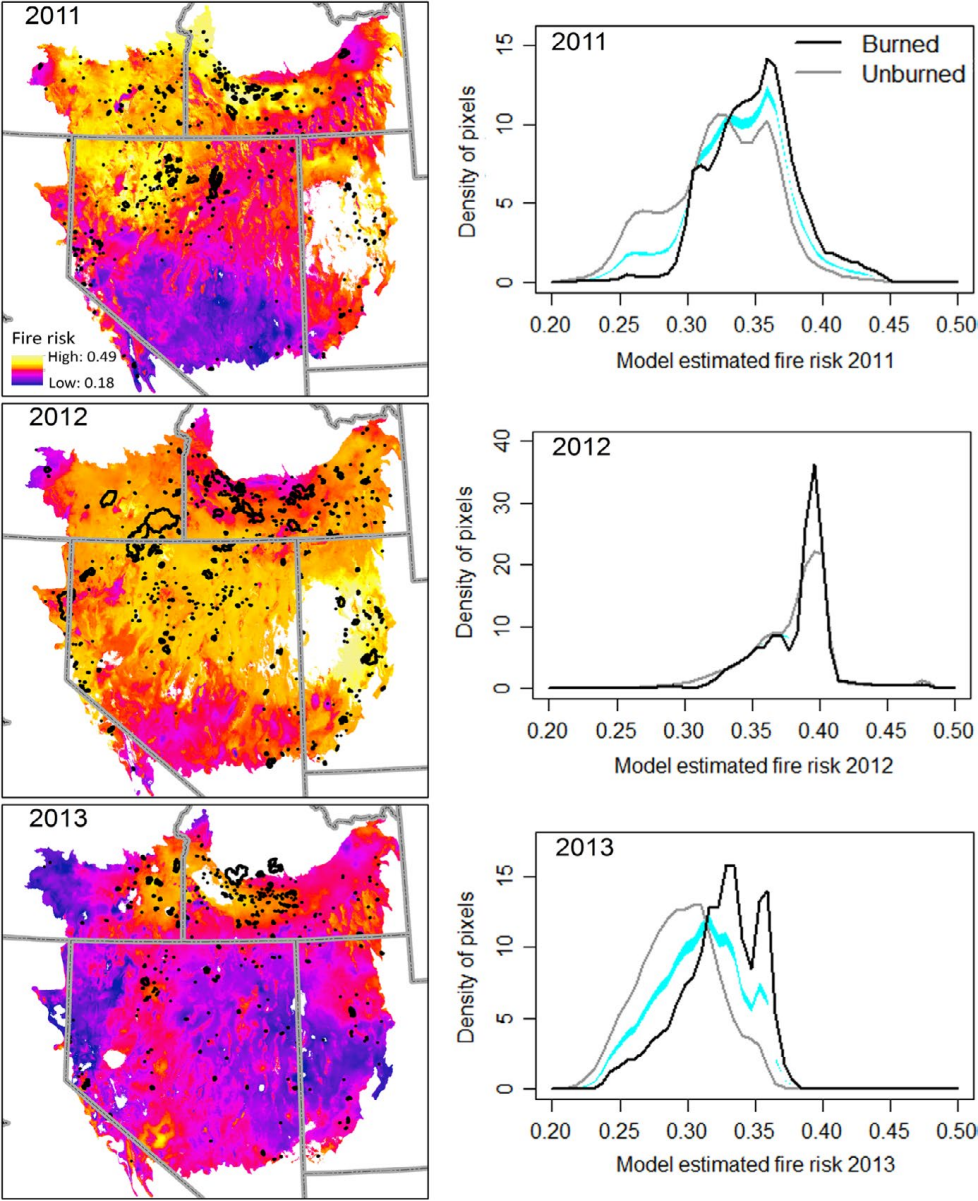
Model predicted elevated fire risk (warmer colors) relative to background levels (cooler colors) for three recent years. Observed fire boundaries (black polygons) are shown for each year. White regions of the Great Basin maps had combinations of predictor variable values that were too rare for reliable predictions of fire risk to be made. Panels at right show, for each year, the probability density of fire risk values for all burned (black lines) and unburned (gray lines) pixels in the Great Basin (*n* = 1.2 million 800‐m pixels total). All three years are plotted on the same *x*‐axis scale for comparison of distributions across time. Blue lines are reference bands indicating the region that the black and gray lines would both occupy if their distributions were not different according to a randomization test produced through 100 bootstrap runs with replacement

Projecting the model to each pixel, based on that pixel's precipitation values for each year 2011–2013, and mapping the resulting fire risk estimates, showed substantial variation in fire risk across the Great Basin within a given year and high temporal variability across years (Figure 12). Fires tended to occur more often in areas of higher model estimated fire risk, and for each of the 3 years examined, the distribution of model estimated fire risk values was significantly different for burned versus unburned pixels (*p *<* *.000 in all three cases). In 2011, fire risk was relatively high in the northwest portion of the Great Basin (i.e., Malheur High Plateau and Humbolt Area MLRAs) and in the Snake River Plains MLRA, but moderate overall in comparison with values from across the Great Basin in 2012. In 2012, fire risk was elevated across a wide swath of the Great Basin, but especially in the Great Salt Lake MLRA. Although much of the study area had elevated risk, fires in 2012 tended to occur in areas at the highest end of the fire risk distribution for that year. The following year, 2013, saw a substantial decrease in relative fire risk across the Great Basin, and areas with elevated risk were limited to the northwest portion of the Owyhee High Plateau and to the Snake River Plains MLRAs. Although 2013 had the greatest difference in fire risk values between burned and unburned pixels of any year examined, model estimated fire risk values for 2013 burned pixels were substantially lower than those that burned in 2012 (Figure 12).

## 4 DISCUSSION

### 4.1 Relationships among weather, herbaceous vegetation, and wildfire

In many arid and semi‐arid ecosystems, precipitation is the main determinant of annual plant productivity (Holmgren et al., 2006). As expected, cheatgrass cover increased in wet years in our focal study area, a finding consistent with many other studies from the western US (e.g., Bradley, 2009; Brummer et al., 2016). Above‐average annual precipitation can result in exceptionally tall cheatgrass that forms dense, continuous cover in the Great Basin (Bradley & Mustard, 2005). Surprisingly, our data also suggest that cheatgrass is responding to multiyear precipitation patterns, despite being an annual plant. This may be the result of increased cheatgrass seed production over several years that then resides in the soil or dense litter (Billings, 1994; Rotundo & Aguiar, 2005). Cheatgrass seed can accumulate quickly in soil (within 1–2 years; Humphrey & Schupp, 2001) and remains viable for more than a decade (Billings, 1994). An experimental study in eastern Oregon found that cheatgrass cover increased suddenly when a wet winter followed 4 years of simulated drought (Bates et al., 2006). However, germination of annuals depends upon available seed and our data suggest that the combination of precipitation in previous years with precipitation in a given year determines cheatgrass cover. In other words, 1 year of high seed production sets the stage for high cheatgrass cover within the next several years as long as annual precipitation is sufficient. High germination rates of cheatgrass during wet years can nearly completely exhaust available stored seed (Smith, Meyer, & Anderson, 2008). Hence, without available seed, cheatgrass would be unable to take advantage of winter and spring moisture, such as when multiyear droughts end (Germino, Belnap, Stark, Allen, & Rau, 2016).

Our data suggest that non‐native forb species dominate when a multiyear dry period transitions to wet conditions. Annual forbs can grow rapidly during brief periods of precipitation, especially at a time when perennial species may be less competitive. This finding is consistent with an experiment in eastern Oregon which demonstrated that annual forb cover peaked when a 2–3 year simulated drought was interrupted by wet winter conditions (Bates et al., 2006). Although subtle, these findings suggest potentially contrasting responses of cheatgrass and non‐native forbs to multiyear precipitation patterns. These non‐native species are competitors in our focal study area, and they may be temporally partitioning resource use. More research on the temporal dynamics between cheatgrass and annual forbs is needed, especially information on how that dynamic influences fuel loads and fire risk.

Native herbaceous vegetation is also an important fuel for wildfires, yet few studies have examined how weather influences the temporal dynamics of native vegetation in the context of fine fuel loads in the Great Basin. We found that cover of native herbs increased when at least one of the previous two winter and springs were dry and particularly in a year that transitioned from dry to wet. Our data suggest that this transition may benefit native herbs because of reduced competition with cheatgrass that depends on early season moisture for germination and growth and does poorly during extended drought. Deep‐rooted native perennials can survive when surface soils dry, but annuals cannot. Also, the opposite is likely true, whereby wet winters and springs increase competition between native herbs and non‐native grasses and forbs that take advantage of shallow soil moisture for increased growth (cover) and seed production (Smith et al., 2008). The distinct influence of weather patterns on cheatgrass versus native plants in the Great Basin was also noted by Bradley and Mustard (2005), who reported that, based on satellite‐derived greenness indices, ecosystems dominated by cheatgrass show an amplified interannual response to rainfall distinct from native bunchgrasses.

Litter is a relatively understudied component of fine fuels in the Great Basin as well (Germino et al., 2016). In our focal study area, litter peaked after 1–2 wet winters and a year after a dry summer, a pattern consistent with our cheatgrass weather model. Cheatgrass cover the previous year was one of best predictors of litter cover in our focal study area. However, cheatgrass was not the only contributor to litter because non‐native forb cover 1 and 2 years before were also important predictors of litter cover. The contribution of non‐native annual forbs as litter, and thus as components of fine fuel accumulation over several years needs additional investigation.

Litter dynamics are complicated to model because litter can accumulate and persist over several years, as well as diminish from decomposition, grazing, redistribution (e.g., from wind), and fire. We found that plant cover, regardless of functional group, three years prior to a given year was a poor predictor of litter cover in that year, which suggests that most fine fuel litter persists for only 1–2 years in this landscape. Factors contributing to litter decomposition in arid environments involve temperature and moisture, including microbial degradation at night, when dew and water vapor are absorbed by litter, and photochemical and thermal degradation during the day (Gliksman et al., 2017). In dry areas, these processes are fairly slow, leading to multiyear persistence and accumulation of fine fuels, especially where non‐native annual grasses and forbs have invaded and domestic grazers are absent (Davies, Svejcar, & Bates, 2009). Rodents and other wild herbivores can also reduce litter accumulation (St Clair, O'Connor, Gill, & McMillan, 2016). However, herbivory and decomposition of litter, particularly cheatgrass litter, may be slowed as cellulose and lignin form an increasing component of above‐ground plant material under increasing atmospheric carbon dioxide levels (Ziska, Reeves, & Blank, 2005).

The development of fine fuels, in combination with natural (i.e., lightning) and human‐caused ignitions, drives wildfires in arid and semi‐arid environments (Crimmins & Comrie, 2004; Greenville, Dickman, Wardle, & Letnic, 2009; Knapp, 1998; Turner, Ostendorf, & Lewis, 2008; Westerling, Gershunov, Brown, Cayan, & Dettinger, 2003). However, in our semi‐arid focal study area, we were unable to predict the number of fires from the cover of herbaceous vegetation and litter. This lack of association may be the result of a high number of human‐caused fires in the area (i.e., outside of a major metropolitan area), especially in years when fuel loads and conditions were not conducive to fire spread. Alternatively, this lack of association could arise because the number of fires in a year is more strongly related to weather patterns than to fuel conditions, a supposition, that is supported by our finding a strong association between weather and number of fires in the same study area. In contrast, we were able to predict the area burned annually, including predicting the probability of unusually large fire years, from fine fuel data. Our data suggest that fire size in this region was mostly a function of growth of native perennial bunchgrasses the year prior to a fire as well as litter from previous year's production of annuals (i.e., cheatgrass and non‐native forbs), but not high cheatgrass cover.

Cheatgrass cover may not be a good predictor of area burned in a given year because the conditions that promote growth of cheatgrass (i.e., high precipitation) may also cause high fuel moisture and thus limit fire spread and area burned. An alternative explanation is that domestic grazing may remove cheatgrass preferentially in wet years when it is abundant and nutritious, thus decoupling the relationship between cheatgrass and area burned in a given year. Experimental research in the northern Great Basin has shown that targeted grazing can reduce above‐ground biomass of cheatgrass by 80%–90% and limit the ability of fire to spread (Diamond, Call, & Devoe, 2010). Winter and spring grazing by sheep and cattle may have influenced the annual wildfire trends in our focal study area, and we consider this an important component of the unexplained variance in our findings.

Although the drying of fuels is important for combustion, temperature variables did not come out as important predictors of fire over the 26‐year period in our focal study area. The lack of temperature effects is puzzling, but perhaps the northern Great Basin is nearly always warm enough to sufficiently cure fine fuels between precipitation events (Davies & Nafus, 2013). Alternatively, our seasonal temperature variables derived from monthly mean values may have been too coarse to predict biological phenomena. However, other studies also have found minimum influence of temperature on cheatgrass cover, with the exception of relatively weak effects of warmer winters and warmer summers (Bradley, 2009; Brummer et al., 2016).

In spite of the apparent disconnect between cheatgrass production and wildfires in any given year, our models are consistent with analyzes of wildfire across the Great Basin that show a 1‐year lag effect of precipitation on both area burned and number of fires (Littell et al., 2009), especially in landscapes dominated by cheatgrass (Balch et al., 2013; Knapp, 1998). Similar to our findings, Balch et al. (2013) found that 27% of the variation in fire size across the Great Basin was a function of annual precipitation the previous year, but not the year of the fire. Several years of above‐average precipitation can lead to a buildup of fine fuel (Hsu et al., 2012; Rao & Allen, 2010) and thus more fires and larger area burned. The accumulation of fine fuels that appears to be driving wildfire patterns in this landscape over several years also suggests that decomposition of litter from grasses and forbs is relatively slow. More research on how Great Basin fires are influenced by the accumulation, persistence, and decomposition or removal of herbaceous litter is needed.

The indirect relationship between antecedent precipitation and fire is well established for arid and semi‐arid ecosystems (e.g., Abatzoglou & Kolden, 2013; Balch et al., 2013; Billings, 1994; Brooks & Matchett, 2006; Crimmins & Comrie, 2004; Knapp, 1998; Littell et al., 2009; Westerling et al., 2003). However, we found that this relationship is far more complex than expected when timing and lag effects are considered. Perhaps the most striking finding from these analyzes was that wildfires are more likely and burn more area when several years of wet winters and springs are followed by a dry spring or summer. When dry summers were not preceded by wet years, fires were less common and less area burned. This suggests that fire risk is not always higher during multiyear droughts, a common misconception. Further, our models of antecedent weather explained only about 38% of the variation in area burned in our focal study area and 43% across the Great Basin, which is slightly higher than the 27% reported by Balch et al. (2013), but also indicates that these processes are complex and influenced by a number of unmeasured factors. Other factors that could influence this relationship include a lack of ignitions in otherwise fire‐prone years, acute fire weather events that cause “blow ups” that lead to unusually large fire size (Barbero, Abatzoglou, Steel, & Larkin, 2014), and fire suppression efforts that keep fire sizes smaller than they might have become. We found that from weather data across the Great Basin we could predict the number of fires that burned in any given year (69% variance explained) better than the area burned (43% variance explained), perhaps because fire starts do not depend as much on fuel conditions and fire suppression as does area burned.

Our models also revealed regional variability in the relationship between precipitation and fire across the Great Basin. This regional variability is probably a reflection of the heterogeneity of topography, climate, soils, vegetation, and land use (Knapp, 1998; Littell et al., 2009). For example, we observed fewer wildfires over the last 35 years in drier, more southerly Great Basin MLRAs, such as the Southern Nevada Basin and Range, which is not nearly as invaded by non‐native annuals as the northern Great Basin (see Appendix 1: Fig. Fig. A2a,b). In these and adjacent hot desert environments, wildfire events are particularly rare historically because of the lack of fuel to carry fire (Brown & Minnich, 1986; Brooks & Berry, 2006; Brooks & Matchett, 2006; Rao & Allen, 2010; Schmid & Rogers, 1988). Research from other hot deserts has shown that at least two years of above‐average precipitation may be required to buildup enough fuel to carry wildfires (Turner et al., 2008). Some researchers estimate that twice the normal rainfall may be required to meet this threshold (Greenville et al., 2009; Letnic & Dickman, 2006).

The fire return interval or time between consecutive fires for a particular location is obviously a critical part of the grass–fire cycle, but we chose not to model these response variables for the Great Basin. Definitions for these variables are scale dependent and further complicated by fire size, shape, and mosaicking (i.e., unburned patches within burn areas). Fires appear to have been fairly uncommon in sagebrush ecosystems historically, with fire return intervals estimated at anywhere from every 1–2 decades up to a century, depending on elevation, location, and site characteristics (Baker, 2006; Heyerdahl, Miller, & Parsons, 2006; Mensing, Livingston, & Barker, 2006). However, recent analyzes have found that fire return intervals are now 2–4 times more frequent where cheatgrass is dominant (Balch et al., 2013). In light of our findings, future research may want to include assessments of how changes in non‐native forbs may also be contributing to fire regime change in the Great Basin. In addition, considerations of future changes in litter accumulation and degradation rates under different climate forecasts may be warranted.

### 4.2 A spatially explicit wildfire risk assessment based entirely on precipitation data

Using precipitation patterns to predict fire risk is intriguing and has been considered for many years, although never implemented (e.g., Hessl, McKenzie, & Schellhaas, 2004; Knapp, 1998; Westerling et al., 2003). For example, Greenville et al. (2009) suggested that assessing cumulative rainfall for 2 years along with rainfall in any given year could be used to successfully predict annual area burned when combined with the mean Southern Oscillation Index from June to November in the year prior. We found that seasonal precipitation information could be applied spatially, such that within a given year, specific locations of the Great Basin could be identified as having higher or lower relative fire risk. Similar to Greenville et al. (2009), we found that precipitation in the year of a fire explained only part of the variation in the number of wildfires or area burned. Fire risk was also associated with seasonally specific precipitation in the years preceding a fire (especially winter and spring precipitation) as well as the change in precipitation in the year of fire relative to previous years. This combined effect of antecedent and current‐year precipitation could allow for better planning of fire suppression or fuel treatments using readily available, existing weather data. Our predictive model could be modified to help resource managers identify areas of high wildfire probability just prior to the fire season or during fuel treatment planning, and thus allocate suppression or fuel treatment resources more effectively. This may be particularly important in light of findings suggesting that fire suppression and fuel treatments are likely to be a more effective means of conservation than postwildfire restoration efforts in sagebrush ecosystems, which have had limited success in many fire‐prone climate zones over the last two decades (Knutson et al., 2014).

Future climate scenarios add a level of uncertainty to many of the processes discussed here (McKenzie, Gedalof, Peterson, & Mote, 2004). For example, increased temperatures could increase evapotranspiration and aridity resulting in less favorable conditions for cheatgrass (Bradley et al., 2016). However, climate change predictions for greater amounts of fall, winter and early‐spring precipitation (Abatzoglou & Kolden, 2011), may create even greater cheatgrass build up (Boyte, Wylie, & Major, 2016). Increasing atmospheric carbon dioxide levels may also exacerbate the increased productivity of cheatgrass, as well as native perennial grasses, and thus increase fine fuel development (Chambers et al., 2014; Smith, Huxman, Zitzer, & Charlet, 2000; Ziska et al., 2005). The uncertainty of these processes may be unsettling to fuel planners and resource managers, but wildfire prediction from current and future climate data is an active area of research (Higuera, Abatzoglou, Littell, & Morgan, 2015; McKenzie & Littell, 2017).

## 5 CONCLUSIONS

Our data supported the hypothesis that precipitation influences herbaceous plant production and thus the amount of fine fuels available for combustion. It is well established that non‐native annual grasses in the Great Basin dry into dense, continuous stands of dead plants and litter, which can fuel wildfires (Brooks et al., 2004; Davies & Nafus, 2013), particularly in the absence of grazing (Davies, Bates, Svejcar, & Boyd, 2010). However, we found that both native and non‐native grasses and forbs influence the number of fires and area burned suggesting that cheatgrass is not the sole driver of the grass–fire cycle in this region. Second, we found support for our hypothesis that years with more fires and area burned tend to occur after one or more years of above‐average precipitation. This could be explained by the accumulation of persistent litter, which increases fuel loads through time. This suggests that precipitation patterns may act indirectly on fire through mechanisms of vegetation and litter production and these indirect effects may be time lagged by 1–3 years as fine fuels accumulate and conditions are right for combustion and fire spread.

These findings have broad implications for conservation and management of the Great Basin as well as arid and semi‐arid shrublands worldwide (D'Antonio & Vitousek, 1992; Hoekstra, Boucher, Ricketts, & Roberts, 2005). The Great Basin has garnered national attention in the U.S. as a result of conservation efforts for the Greater Sage‐grouse (*Centrocercus urophasianus*). One of the greatest threats to this iconic bird is the loss of shrubland habitat due to the cheatgrass–fire cycle that has converted native shrublands to annual grasslands after burning repeatedly. This threat is so significant that in 2015 the Department of Interior released Secretarial Order (SO 3336) to enhance policies and strategies for preventing and suppressing rangeland fire and restoring rangeland landscapes affected by fire in the western United States (USDI, 2015). The major findings from our analyzes span the majority of the western range of the Greater Sage‐grouse and the application of our precipitation‐based fire risk model could be an important contribution to meeting the Secretarial Order. This information may also help resource managers prioritize the location and timing of fuel management actions, such as maintenance of fuel breaks, green stripping, brown stripping, and targeted grazing (Maestas et al., 2016). Our findings suggest that land managers interested in reducing fine fuels may need to consider how to manage previous years’ herbaceous production, in the form of litter from annual forbs and cheatgrass, instead of solely managing biomass from the current growing season.

## CONFLICT OF INTEREST

None declared.

## Figures and Tables

**Figure A2A:**
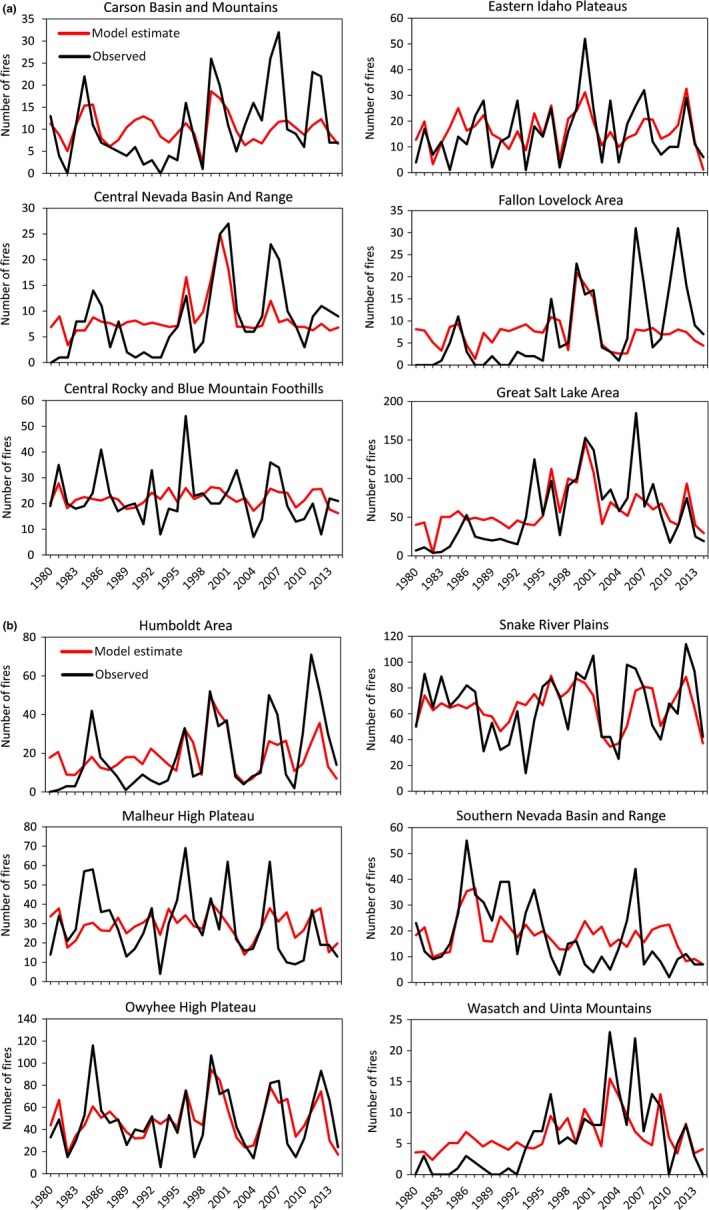
Observed (black lines) and model estimated (red lines) number of fires for each year by MLRA. Model estimates are derived from precipitation variables in the Great Basin‐level model. Note different *y*‐axes scales

**Figure A2B:**
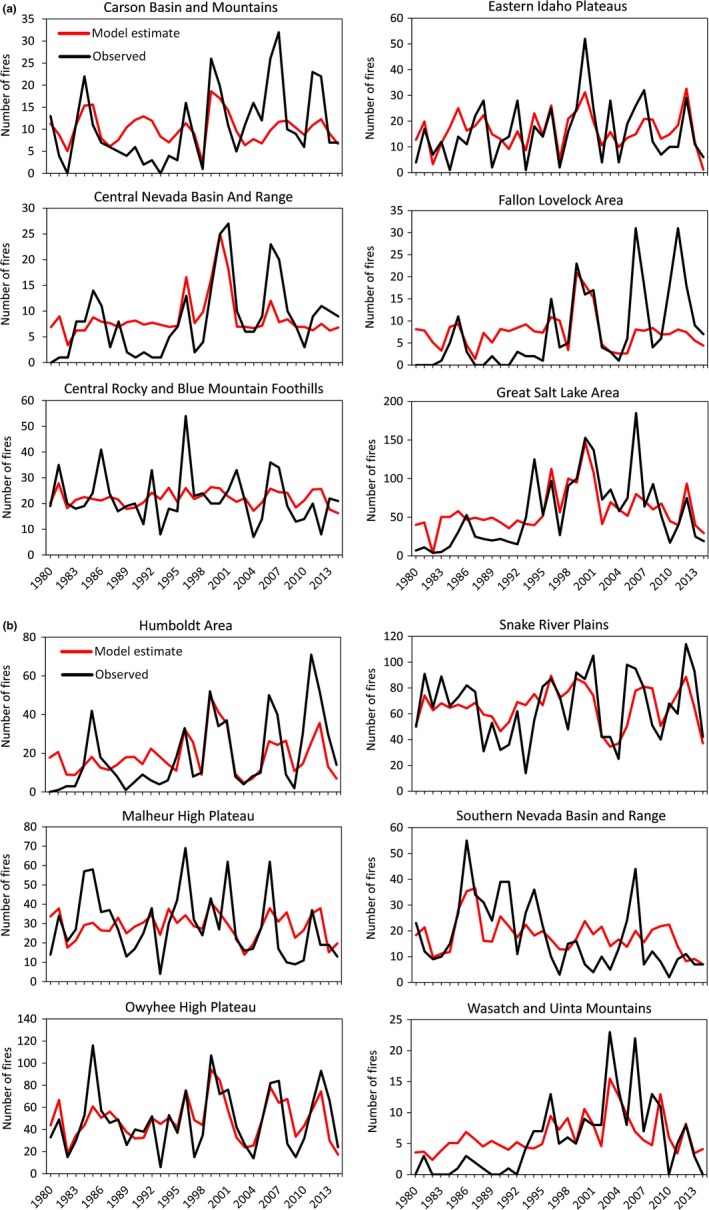
Observed (black lines) and model estimated (red lines) number of fires for each year by MLRA. Model estimates are derived from precipitation variables in the Great Basin‐level model. Note different *y*‐axes scales

**Figure A3A:**
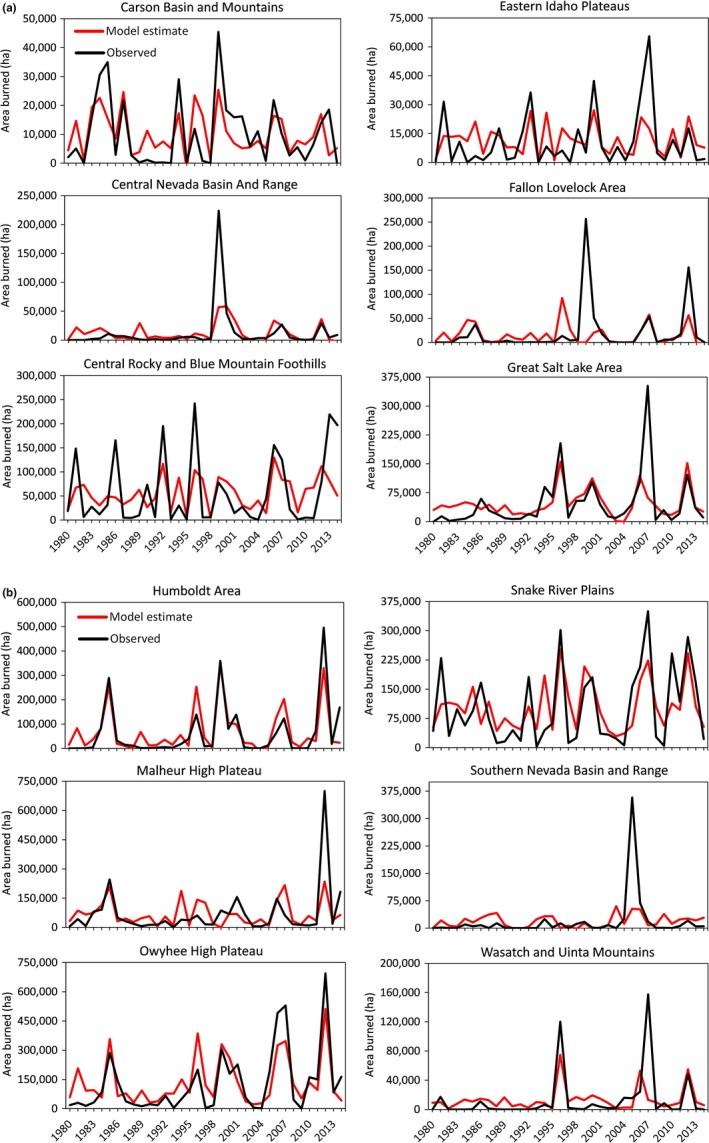
Observed (black lines) and model estimated (red lines) area burned for each year by MLRA. Model estimates are derived from precipitation variables in the Great Basin‐level model. Note different *y*‐axes scales

**Figure A3B:**
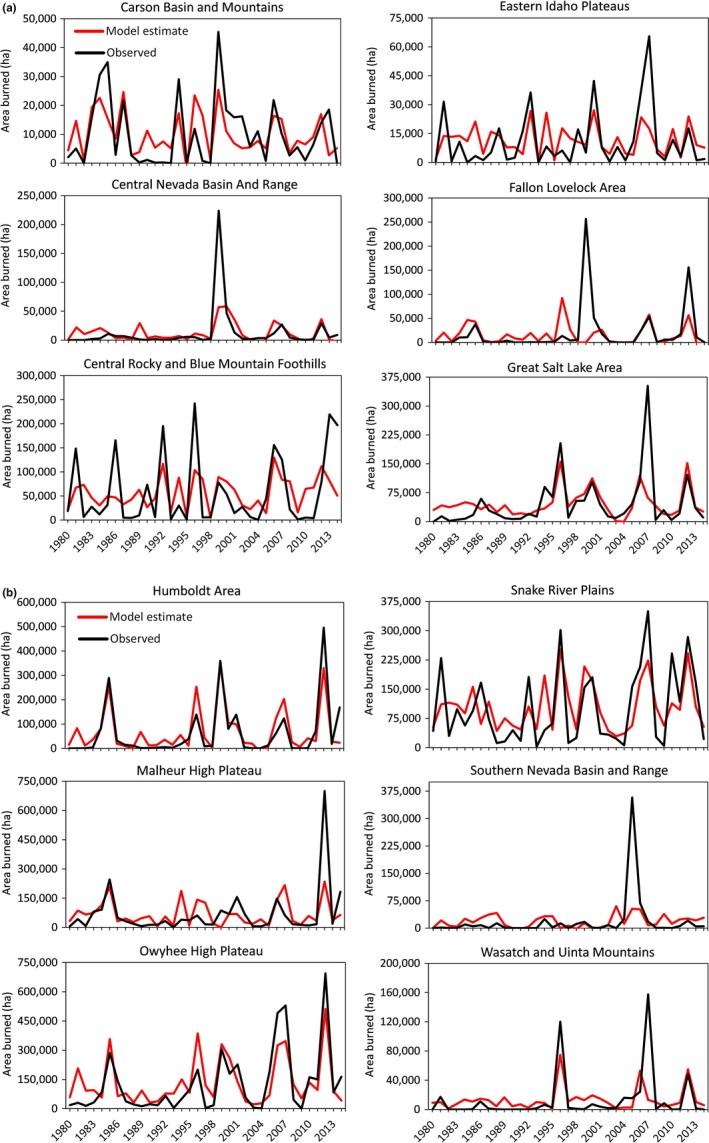
Observed (black lines) and model estimated (red lines) area burned for each year by MLRA. Model estimates are derived from precipitation variables in the Great Basin‐level model. Note different *y*‐axes scales

## APPENDIX A

### A.1

**Table A1:** List of vegetation classes in the Great Basin derived from LANDFIRE (http://www.landfire.gov/) ordered from largest to smallest area. Vegetation classes are composites of potential vegetation types, which are biophysical characteristics of the Great Basin

Vegetation class	Variable name	Hectares	Percent of great basin (%)
Wyoming sagebrush shrubland	WYSAGE	1,358,303	26
Salt desert shrubland	SDS	1,206,619	23
Other vegetation types	OTHER	1,132,780	21
Juniper woodland	Juniper	969,701	18
Dwarf sagebrush shrubland	DWSage	449,497	8
Mountain sagebrush shrubland	MTSAGE	189,121	4
		5,306,020	100
